# A bioinformatics approach to the identification of novel deleterious mutations of human TPMT through validated screening and molecular dynamics

**DOI:** 10.1038/s41598-022-23488-z

**Published:** 2022-11-07

**Authors:** Sidharth Saxena, T. P. Krishna Murthy, C. R. Chandrashekhar, Lavan S. Patil, Abhinav Aditya, Rohit Shukla, Arvind Kumar Yadav, Tiratha Raj Singh, Mahesh Samantaray, Amutha Ramaswamy

**Affiliations:** 1 Department of Biotechnology, Ramaiah Institute of Technology, Bengaluru, Karnataka 560054 India; 2 Department of Biotechnology and Bioinformatics, Jaypee University of Information Technology (JUIT), Solan, Himachal Pradesh 173234 India; 3 Department of Bioinformatics, Pondicherry University, Pondicherry, 605014 India

**Keywords:** Computational biology and bioinformatics, Literature mining

## Abstract

Polymorphisms of Thiopurine S-methyltransferase (TPMT) are known to be associated with leukemia, inflammatory bowel diseases, and more. The objective of the present study was to identify novel deleterious missense SNPs of TPMT through a comprehensive in silico protocol. The initial SNP screening protocol used to identify deleterious SNPs from the pool of all TPMT SNPs in the dbSNP database yielded an accuracy of 83.33% in identifying extremely dangerous variants. Five novel deleterious missense SNPs (W33G, W78R, V89E, W150G, and L182P) of TPMT were identified through the aforementioned screening protocol. These 5 SNPs were then subjected to conservation analysis, interaction analysis, oncogenic and phenotypic analysis, structural analysis, PTM analysis, and molecular dynamics simulations (MDS) analysis to further assess and analyze their deleterious nature. Oncogenic analysis revealed that all five SNPs are oncogenic. MDS analysis revealed that all SNPs are deleterious due to the alterations they cause in the binding energy of the wild-type protein. Plasticity-induced instability caused by most of the mutations as indicated by the MDS results has been hypothesized to be the reason for this alteration. While in vivo or in vitro protocols are more conclusive, they are often more challenging and expensive. Hence, future research endeavors targeted at TPMT polymorphisms and/or their consequences in relevant disease progressions or treatments, through in vitro or in vivo means can give a higher priority to these SNPs rather than considering the massive pool of all SNPs of TPMT.

## Introduction

Human Thiopurine S-methyltransferase (TPMT) is a protein-coding gene on chromosome 6p22.3 that codes for the enzyme Thiopurine *S*-methyltransferase. It is a monomer that belongs to the class I-like SAM-binding methyltransferase superfamily and is composed of 245 amino acid residues. This cytosolic enzyme catalyzes the S-methylation of thiopurines such as azathioprine, 6-thioguanine, 6-mercaptopurine, etc. Thiopurines were originally developed in the 1950s for the treatment of acute myeloid leukemia in children by Gertrude Elion and George Hitchings^1^, which eventually won them the Nobel Prize in Physiology or Medicine in 1988. These thiopurine drugs are antimetabolites and immunomodulators and are used extensively as anticancer and immunosuppressive agents in the treatment of rheumatoid arthritis, inflammatory bowel diseases (IBD) including Crohn’s disease and ulcerative colitis, acute lymphoblastic leukemia (ALL), and other autoimmune disorders. Although about two-third of patients do not suffer from any notable side effects throughout their treatments, the rest often cannot continue their treatments or have their treatments greatly modified due to cytotoxic side effects and an increased risk of malignancies. Most patients with thiopurine-induced hepatotoxicity have a high concentration of 6-methylmercaptopurine ribonucleotide or 6-MMPR (formed by TPMT) one week after treatment initiation. There is also evidence that elevated levels of 6-methylmercaptopurine or 6-MMP (formed by TPMT) are correlated with hepatotoxicity^2^. To address these side effects, improved drug delivery mechanisms such as nano-formulations and delayed-release tablets are in development, none of which are currently in clinical practice^3^.

Akin to many immunosuppressants, the mechanism of action of thiopurines involves the inactivation of critical T-cell processes leading to inflammation. Unfortunately, the exact mechanism of action of thiopurines and their effects are not yet comprehensively understood. A combination of many different factors has been proposed for their cytotoxicities, such as the inhibition of de novo purine synthesis (DNPS), alterations in DNA methylation state, disruption of guanosine-triphosphate (GTP) signaling, and the incorporation of thioguanine nucleotides (TGN) as bases into DNA. The incorporation of thioguanine nucleotides into DNA causes the induction of apoptosis^4^.

Around 99.9% of the DNA sequence is identical between any two human genomes selected at random. The variation in 0.1% of the DNA sequence comprises genetic alterations known as polymorphisms. One such form of these polymorphisms involves the alteration of one nucleotide and is aptly referred to as a single nucleotide polymorphism (SNP). Nearly 90% of all human DNA polymorphisms are attributed to SNPs and hence, they are by far the most common form of genetic variation that occurs in the human genome^5^. In fact, an SNP occurs once every 300 base pairs on average in the human genome^6^. SNPs can result in a change of the encoded amino acids if they are non-synonymous or can be silent if they are synonymous or simply occur in the non-coding regions without consequence. Non-synonymous SNPs (nsSNPs) can alter the function of the protein products of genes if they occur in the coding regions, which can be especially dangerous if the gene holds biological significance^7^.

One of the major reasons for the cytotoxic side effects of thiopurine drugs is TPMT deficiency in patients and most TPMT polymorphisms often reduce TPMT activity^8^. Furthermore, it has already been established that several alleles of TPMT are in fact, disease-associated with respect to leukemia^9–13^, IBD^14–19^, and more. Such polymorphisms can also severely affect protein stability^20^ and cause adverse drug reactions^21^. Therefore, SNPs of TPMT can be crucial in the disease progression and treatments associated with leukemia, IBD, and more. This warrants the identification of novel SNPs of TPMT which are deleterious in nature. While the conduction of in vitro and/or in vivo methods for the identification of novel deleterious nsSNPs from the massive pool of all nsSNPs is more conclusive than in silico methods, they are often challenging and expensive protocols. As a result, the present work is focused on the identification of novel deleterious missense mutations of TPMT through in silico means. These SNPs can later be given a higher priority by future researchers interested in identifying TPMT variants and their potential consequences through in vitro and/or in vivo means*.* Although to validate the initial in silico screening process, deleterious SNPs of TPMT identified through in vitro or in vivo means were also subjected to this screening process. The screening process was able to accurately identify a significant percentage of these pre-identified SNPs, as discussed in “Screening of nsSNPs” section.

Similar in silico studies have been conducted for several genes in the past to understand the molecular mechanisms of disease-causing mutations^22–28^. In fact, an in silico study for TPMT was also carried out in the past^29^, but that study only considered a few established mutations and did not aim to find novel deleterious SNPs of TPMT. It also considered far fewer tools for the in silico protocol than the present study. Meanwhile, the present study aimed at identifying novel deleterious missense nsSNPs by screening all missense nsSNPs of TPMT listed in the dbSNP database in an attempt to identify those SNPs from the database that are extremely deleterious to both the structure and the natural functioning of the protein. The usage of computational tools for the classification of variants is at the heart of an in silico based approach for the identification of novel deleterious mutations. The tools chosen for this study were based on the variety of techniques used by the various screening tools in order to arrive at an unbiased consensus-based result with respect to the classification of deleterious TPMT variants. The screened SNPs were then subjected to further in silico analyses such as conservation analysis, interaction analysis, oncogenic analysis, phenotypic analysis, structural analysis, and post-translational modification (PTM) analysis.

Molecular dynamics simulations (MDS) simulate the motions of every single atom in a protein or any other molecular system over time-based on the classical equations of motion governing interatomic interactions. Several important biomolecular processes, such as protein folding, ligand binding, and conformational change can be studied through MDS. Most importantly, they can simulate how a particular biomolecule will respond to mutations^30,31^. Hence, MDS was also performed in order to assess the impact of the mutations with respect to the wild-type protein at an atomic level and from a dynamic perspective as opposed to the static protocol of other structural analysis tools. Several of the aforementioned in silico studies have also employed MDS to gain a greater understanding of the deleterious nature of the screened mutations. Finally, a consensus-based result was obtained which is at the heart of the in silico pipeline of the present study. Apart from this, two other factors make the present study unique. Firstly, the utilization of a consensus-based approach for the screening of deleterious mutations is based on a lot of different computational tools that possess different pipelines to predict the nature of SNPs. Secondly, the validation of this in silico screening pipeline by applying it to the SNPs associated with TPMT alleles that have already been proven to be deleterious by experimental methods in prior research efforts and assessing how many of them are redetected through this pipeline. Results showed that five novel mutations, namely W33G, W78R, V89E, W150G, and L182P can have a deleterious effect on TPMT and that W33G is the most deleterious among them.

### Materials and methods

### Collection of data

**Figure 1:**
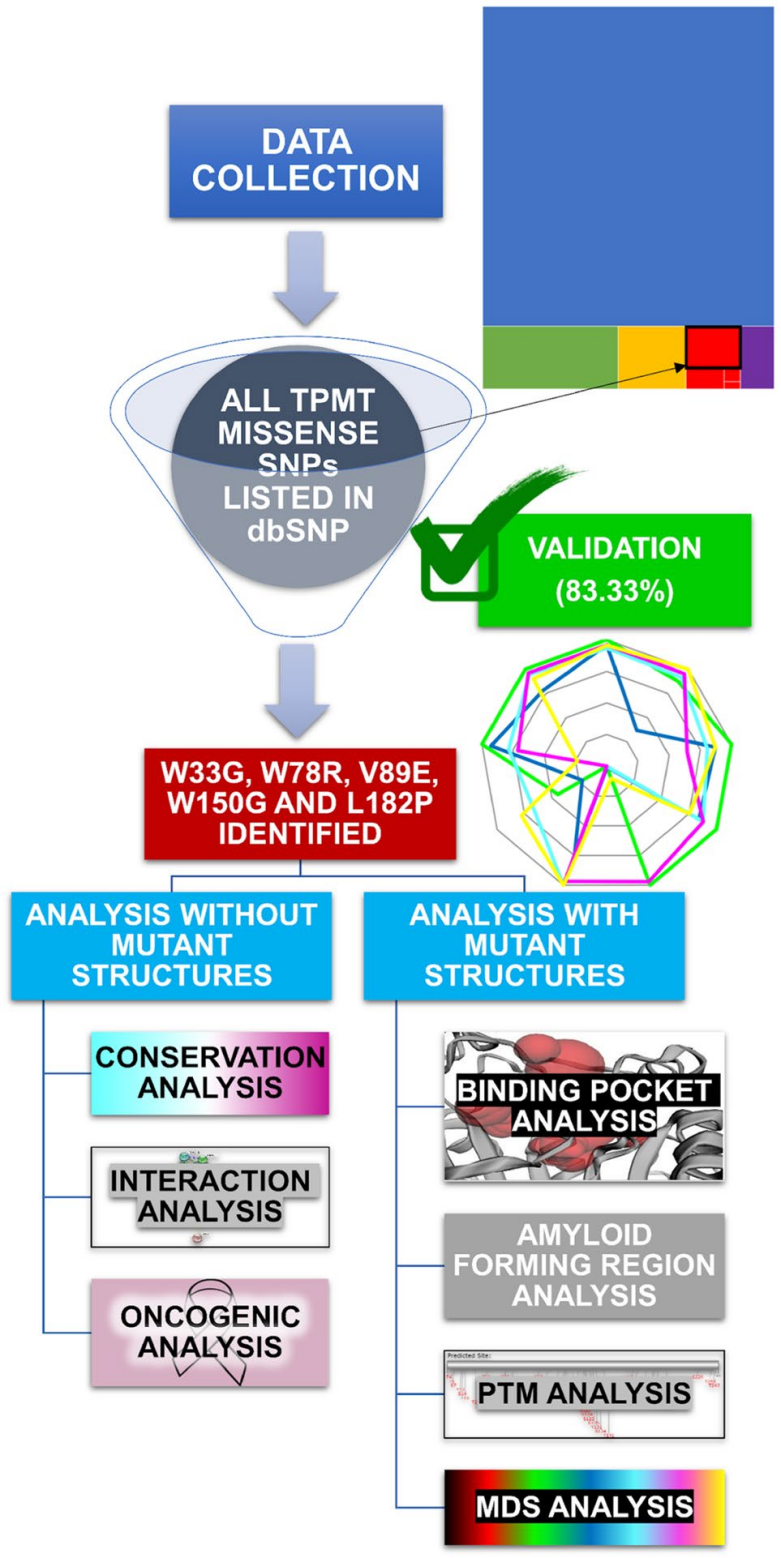
Visual representation of the methodology followed in the present study (The rectangle on the top right represents the pool of all TPMT SNPs. Please refer to Figure S1 for more details).

The NCBI dbSNP database was utilized to obtain the missense nsSNPs of the human TPMT gene (access date: July 26, 2021)^32^. dbSNP database contains more than 650 million human SNPs (Build 152, November 2018) and includes clinically relevant SNPs from the ClinVar database as well. The UniProt database was used to obtain the sequence for the same (UniProt ID: P51580)^33^. The structure of the TPMT protein with PDB ID 2H11^34^ was obtained through the Protein Data Bank. The overall protocol followed in the present study has been visually presented in Fig. 1.

### Screening of SNPs through sequence-based tools

12 sequence-based tools were utilized to analyze the functional consequences of the missense nsSNPs of TPMT, including Protein Variation Effect Analyzer (PROVEAN)^35^, Protein Analysis Through Evolutionary Relationships (PANTHER)^36^, Polymorphism Phenotyping v2 (PolyPhen-2)^37^, Mutation Assessor^38^ and Sorting Intolerant From Tolerant (SIFT)^39^. Apart from these tools, consensus-based tools such as Meta-SNP^40^ and PredictSNP1^41^ were utilized in the screening process as well. Furthermore, SuSPect^42^, PMut^43^, SNAP2^44^, PhD-SNP^45^ and SNPs&GO^46^ were used as well during the screening process.

### Screening of SNPs through structure-based tools

The stability changes imparted on the protein structure as a consequence of the nsSNPs identified to be dangerous (by at least nine out of the 12 aforementioned sequence-based tools), were assessed using structure-based tools. Eight structure-based tools were used, including CUPSAT^47^, DUET^48^, I-Mutant 3.0^49^, MUpro^50^, INPS-MD^51^, PoPMuSiC^52^, HoTMuSiC^53^ and SNPMuSiC^54^. The melting point of TPMT was inputted as 319.95 K for HoTMuSiC^55^. Furthermore, for the screening process, an SNP was considered deleterious by the three MuSiC tools only if the SNP was predicted to “Strongly Decrease” the stability of the protein (A prediction score close to or greater than 4, close to or lesser than -10 and close to or greater than 1 for PoPMuSiC, HoTMuSiC and SNPMuSiC, respectively imply that the particular SNP will “Strongly Decrease” the protein stability). This was done in order to identify the most deleterious missense SNPs among all of the missense SNPs.

### Validation of the screening pipeline

Many SNPs of TPMT have already been proven to be deleterious by prior research efforts through experimental means. To validate and assess the in silico screening pipeline utilized in this study, the same screening methodology was applied for these pre-identified SNPs as well, albeit with one small difference. The difference is that the SNPs predicted to “Decrease” protein stability by the three MuSiC tools were considered deleterious, as opposed to the ones predicted to “Strongly Decrease” the protein stability. This was done because, in the validation phase, SNPs already proven to be deleterious were to be re-identified rather than finding the most deleterious SNPs among them.

### Conservation analysis

The evolutionary conservation analysis of TPMT residues was conducted through the ConSurf server^56^. Furthermore, multiple sequence alignment (MSA) was performed through various tools including Clustal Omega, Kalign, MUSCLE, T-Coffee and MAFFT^57^ for TPMT sequences belonging to different species including *Homo sapiens* (P51580), *Gorilla gorilla gorilla* (Q3BCR3), *Pan troglodytes* (Q3BCR8), *Pongo abelii* (A0A0B4J2I0), *Rhinopithecus bieti* (A0A2K6KP23), *Chlorocebus sabaeus* (A0A0D9R5L7), *Macaca mulatta* (F6TPF9), *Callithrix jacchus* (F6PNF2), *Vicugna pacos* (A0A6I9HY34) and *Panthera tigris* (Q3BCR2). Apart from MSA tools and ConSurf, NLM’s (National Library of Medicine) CDD (Conserved Domain Database)^58^ was also considered. The “Type Selection” parameter was chosen as “the most diverse members” which identifies the most dissimilar TPMT sequences, as determined from the domain model MSA.

### Interaction analysis

The STRING database^59^ was used to conduct the interaction analysis for TPMT. Interaction analysis is important as it sheds light on the consequences of TPMT mutants on the biological processes that involve the interaction network of TPMT. This is because, in the presence of highly deleterious mutations, the structure and function of TPMT will be impaired. Since all proteins in the interaction network must function appropriately for the associated biological processes to occur in the desired manner, a dysfunctional TPMT may very well hinder or cause a lot of problems for said biological processes.

### Oncogenic analysis and phenotypic analysis

To assess the oncogenicity of the five missense SNPs, CScape^60^, CScape-somatic^61^, and Dr Cancer^62^ were utilized. The phenotypic consequences of the five mutations were predicted using the FATHMM (Functional Analysis through Hidden Markov Models) tool^63^. The “Inherited Disease” section of the tool was utilized, where the weighted algorithm was used as the prediction algorithm. Furthermore, the “Phenotypic Associations” parameter was selected as “Human Phenotype Ontology”.

### Modelling of TPMT mutants and assessing the structural effects of mutations

Modeling of the TPMT mutants (W33G, W78R, V89E, W150G, and L182P) was performed by using the PyMol software^64^. The HOPE (Have Your Protein Explained) server^65^ was used to visualize the mutations and also to gain some insight into the structural consequences caused by the mutations. NetSurfP-2.0^66^ was used to identify alterations in the secondary structure of the protein that are caused by the five deleterious mutations. It utilized the amino acid sequences of the wild-type protein and mutant proteins to generate the results. Furthermore, the alterations caused to the binding pockets of the wild-type protein were identified using the CASTp 3.0 server^67^. AmylPred 2^68^ was also used for identifying amyloid-forming regions that may be associated with several conformational diseases, called amyloidoses. Furthermore, MDS analysis was performed to validate and expand upon the structural analysis.

### Post-translational modification analysis

The Group-based Prediction System (GPS) 5.0 software^69^ was used for PTM analysis which can predict the PTM sites within a protein. This software was used to predict the tyrosine kinase phosphorylation sites along with the serine/threonine kinase phosphorylation sites of TPMT.

### Molecular dynamics simulations

The crystal structure of human TPMT (2H11) was subjected to MDS through GROMACS version 2019.4^70^. The GROMOS9653a6 force field was used for the generation of the protein parameters. The gmx editconf tool was used to build the cubic simulation box. The steepest descent algorithm was used to vacuum minimize the processed setup for 1500 steps. The simple point-charge (SPC) water model was used to perform solvation by using the gmx solvate tool. The system was electro-neutralized through the gmx genion tool, following which the steric clashes were removed. After this, energy minimization was performed to optimize the structure. System equilibration was performed through NVT equilibration and NPT ensemble. 1 ns of NVT equilibration was performed where the system was heated up to 300 K, to stabilize the temperature of the system. Furthermore, 1 ns of NPT ensemble was performed to stabilize the pressure and density of the system. After system equilibration, each structure was subjected to MDS over a simulation time of 100 ns.

As mentioned in the introduction section, MDS can be used to analyze the consequences of mutations at an atomic level through dynamic simulations of protein structure alterations. The MDS parameters including Root-mean-square deviation (RMSD), Root-mean-square fluctuations (RMSF), radius of gyration (Rg), solvent-accessible surface area (SASA) and intramolecular hydrogen bonding help us understand various aspects of protein structure alterations such as structural alterations in flexibility, rigidity, compactness and more. Trajectory analysis of the wild-type protein and the five mutant proteins was done using various GROMACS analysis tools. RMSD, RMSF, Rg, SASA and intramolecular hydrogen bonding, were calculated using the gmx rms, gmx rmsf, gmx gyrate, gmx sasa and gmx hbond tools, respectively. Secondary structure analysis (SSA) was performed through the do_dssp tool. Principal component analysis (PCA) was performed using the gmx anaeig and gmx covar tools. The Molecular Mechanics Poisson–Boltzmann Surface Area (MM-PBSA) method was utilised to calculate the binding energy and its components including van der Waals, electrostatic energy, polar solvation energy and SASA energy using the g_mmpbsa tool^71^. The relevance and interpretation of all of the aforementioned MDS parameters have been discussed in “Molecular dynamics simulations analysis” section.

## Results and discussion

### SNP distribution

In all, 10,305 SNPs of TPMT were obtained from dbSNP. SNPs in the intronic region made up 83.55% (8610) of all SNPs. 7.64% (787) and 3.86% (398) of the SNPs were present in the 3’UTR and 5’UTR regions, respectively. Furthermore, all forms of coding SNPs accounted for merely 3.06% of all SNPs. Among coding SNPs missense SNPs, synonymous SNPs, frameshift SNPs and nonsense SNPs accounted for 2.07% (213), 0.69% (71), 0.19% (20) and 0.11% (11), respectively. Other SNPs accounted for the remaining 1.89% (195). The present study has considered only missense SNPs of TPMT. The distribution of TPMT SNPs is shown in Figure S1.

### Screening of nsSNPs

All missense SNPs of TPMT present in the dbSNP database were screened using sequence-based and structure-based tools to identify the most deleterious ones among them. Prediction results of all missense SNPs are provided in Table S1 and S2.

For sequence-based screening, the aforementioned 12 sequence-based tools were used, and SNPs found to be deleterious by at least nine out of the 12 tools were considered for further analysis. 53 such SNPs were found, which are reported in Table 1. These 53 missense SNPs were then subjected to structure-based screening using eight structure-based tools. Missense SNPs identified as deleterious by at least six of these tools were considered for further analysis. Six such SNPs were identified including W33G, W78R, V89E, W150G, L182P, and Y240S. However, owing to the fact that Y240S has already been proven to be deleterious^9,19,20^, it was not considered for further analyses. Hence, five novel extremely deleterious missense SNPs of human TPMT were identified at the end of the screening process which was then subjected to further analyses. The results of the structure-based screening are presented in Table 2. The rationale behind considering the deleterious prediction of any 9 sequence-based tools and any 8 structure-based tools, many of which have varying approaches for variant classification is to get an unbiased result. If an SNP is predicted to be deleterious by 9 different sequence-based tools and 8 different structure-based tools, several of which have varying approaches, then the probability of it being dangerous is high.

**Table 1:** List of missense nsSNPs predicted to be deleterious by at least nine out of 12 sequence-based tools.

Variant ID	SNP	PROVEAN	PANTHER	Polyphen-2	Mutation Assessor	Meta-SNP	PMut	PredictSNP1	SNAP2	SuSPect	SNPs&GO	PhD-SNP	SIFT
rs72552741	W33G	Deleterious	Probably damaging	Probably damaging	High	Disease	Disease	Deleterious	Effect	Deleterious	Disease	Disease	Deleterious
rs200695400	F40S	Deleterious	Probably damaging	Probably damaging	High	Disease	Disease	Deleterious	Effect	Deleterious	Disease	Disease	Deleterious
rs1446592306	F40L	Deleterious	Probably damaging	Probably damaging	High	Disease	Disease	Deleterious	Effect	Deleterious	Disease	Disease	Deleterious
rs757081801	H41R	Deleterious	Probably damaging	Possibly damaging	High	Disease	Disease	Deleterious	Effect	Deleterious	Disease	Disease	Deleterious
rs762702707	H46R	Deleterious	Probably damaging	Probably damaging	Medium	Neutral	Disease	Deleterious	Effect	Deleterious	Disease	Disease	Deleterious
rs72552740	L49S	Deleterious	Probably damaging	Probably damaging	High	Disease	Disease	Deleterious	Effect	Deleterious	Disease	Disease	Deleterious
rs757971326	H52D	Deleterious	Possibly damaging	Possibly damaging	Medium	Disease	Disease	Deleterious	Effect	Deleterious	Disease	Disease	Deleterious
rs1236222449	F66I	Deleterious	Probably damaging	Probably damaging	High	Disease	Disease	Deleterious	Effect	Neutral	Neutral	Disease	Neutral
rs1368439131	P68L	Deleterious	Probably damaging	Probably damaging	High	Disease	Disease	Deleterious	Effect	Deleterious	Disease	Disease	Deleterious
rs1784276391	P68S	Deleterious	Probably damaging	Probably damaging	High	Disease	Disease	Deleterious	Effect	Deleterious	Disease	Disease	Deleterious
rs200591577	L69V	Deleterious	Probably damaging	Probably damaging	High	Disease	Disease	Deleterious	Effect	Deleterious	Disease	Disease	Deleterious
rs200591577	L69F	Deleterious	Probably damaging	Probably damaging	High	Disease	Disease	Deleterious	Effect	Deleterious	Disease	Disease	Deleterious
rs777686348	G71R	Deleterious	Probably damaging	Probably damaging	High	Disease	Disease	Deleterious	Effect	Deleterious	Disease	Disease	Deleterious
rs778578091	E75K	Deleterious	Possibly damaging	Probably damaging	Medium	Disease	Disease	Deleterious	Effect	Deleterious	Disease	Disease	Deleterious
rs1200214781	M76K	Deleterious	Probably damaging	Probably damaging	High	Disease	Disease	Deleterious	Effect	Deleterious	Disease	Disease	Deleterious
rs1256618794	W78C	Deleterious	Probably damaging	Probably damaging	High	Disease	Disease	Deleterious	Effect	Deleterious	Disease	Disease	Deleterious
rs753277177	W78R	Deleterious	Probably damaging	Probably damaging	High	Disease	Disease	Deleterious	Effect	Deleterious	Disease	Disease	Deleterious
rs1800462	A80P	Deleterious	Probably damaging	Probably damaging	High	Disease	Disease	Deleterious	Effect	Deleterious	Disease	Disease	Deleterious
rs111901354	R82G	Deleterious	Probably benign	Probably damaging	Medium	Disease	Disease	Deleterious	Effect	Neutral	Disease	Disease	Deleterious
rs111901354	R82W	Deleterious	Probably benign	Probably damaging	Medium	Disease	Neutral	Deleterious	Effect	Deleterious	Disease	Disease	Deleterious
rs1293957844	G83V	Deleterious	Probably damaging	Probably damaging	High	Disease	Disease	Deleterious	Effect	Deleterious	Disease	Disease	Deleterious
rs1235431245	H84D	Deleterious	Probably damaging	Probably damaging	High	Disease	Neutral	Deleterious	Effect	Deleterious	Disease	Disease	Deleterious
rs1582044292	H84L	Deleterious	Probably damaging	Probably damaging	Medium	Disease	Neutral	Deleterious	Effect	Deleterious	Disease	Disease	Neutral
rs753545734	G88S	Deleterious	Probably damaging	Probably damaging	High	Disease	Disease	Deleterious	Effect	Deleterious	Disease	Disease	Deleterious
rs753545734	G88C	Deleterious	Probably damaging	Probably damaging	High	Disease	Disease	Deleterious	Effect	Deleterious	Disease	Disease	Deleterious
rs1784191846	V89E	Deleterious	Probably damaging	Probably damaging	High	Disease	Disease	Deleterious	Effect	Deleterious	Disease	Disease	Deleterious
rs1681788109	E90V	Deleterious	Possibly damaging	Probably damaging	High	Disease	Disease	Deleterious	Effect	Deleterious	Disease	Disease	Deleterious
rs1474060016	L105R	Deleterious	Possibly damaging	Probably damaging	High	Disease	Disease	Deleterious	Effect	Neutral	Disease	Disease	Deleterious
rs886061266	I112N	Deleterious	Probably benign	Probably damaging	Medium	Disease	Disease	Deleterious	Effect	Neutral	Disease	Disease	Deleterious
rs1396619437	S129P	Deleterious	Probably benign	Probably damaging	Medium	Disease	Disease	Deleterious	Effect	Neutral	Disease	Disease	Deleterious
rs72552738	C132Y	Deleterious	Probably damaging	Possibly damaging	Medium	Disease	Disease	Deleterious	Neutral	Deleterious	Disease	Disease	Deleterious
rs72552737	G144R	Deleterious	Probably damaging	Probably damaging	Medium	Disease	Neutral	Deleterious	Effect	Neutral	Disease	Disease	Deleterious
rs1310627040	I149T	Deleterious	Probably damaging	Probably damaging	Medium	Disease	Disease	Deleterious	Effect	Deleterious	Disease	Disease	Deleterious
rs1447033392	W150G	Deleterious	Probably damaging	Probably damaging	Medium	Disease	Disease	Deleterious	Effect	Deleterious	Disease	Disease	Deleterious
rs1354851110	D151Y	Deleterious	Probably damaging	Probably damaging	High	Disease	Disease	Deleterious	Effect	Deleterious	Disease	Disease	Deleterious
rs1408113946	R152T	Deleterious	Probably damaging	Probably damaging	High	Disease	Disease	Deleterious	Effect	Deleterious	Disease	Disease	Deleterious
rs1800460	A154T	Deleterious	Probably benign	Possibly damaging	High	Disease	Disease	Deleterious	Effect	Deleterious	Disease	Disease	Deleterious
rs1158437171	L155S	Deleterious	Possibly damaging	Probably damaging	High	Disease	Disease	Deleterious	Effect	Deleterious	Disease	Disease	Deleterious
rs112339338	R163S	Deleterious	Probably damaging	Probably damaging	High	Disease	Disease	Deleterious	Effect	Deleterious	Disease	Disease	Deleterious
rs112339338	R163C	Deleterious	Probably damaging	Probably damaging	High	Disease	Disease	Deleterious	Effect	Deleterious	Disease	Disease	Deleterious
rs201695576	Y166C	Deleterious	Probably damaging	Probably damaging	High	Disease	Disease	Deleterious	Effect	Deleterious	Disease	Disease	Deleterious
rs1386533390	L182P	Deleterious	Probably damaging	Probably damaging	High	Disease	Disease	Deleterious	Effect	Deleterious	Disease	Disease	Deleterious
rs1783991755	V184D	Deleterious	Probably benign	Probably damaging	Medium	Disease	Disease	Deleterious	Effect	Neutral	Disease	Disease	Deleterious
rs747307984	L185R	Deleterious	Probably benign	Possibly damaging	High	Disease	Disease	Deleterious	Effect	Deleterious	Disease	Disease	Deleterious
rs1554137341	Y187C	Deleterious	Probably damaging	Probably damaging	High	Disease	Disease	Deleterious	Effect	Deleterious	Disease	Disease	Deleterious
rs758437011	G194S	Deleterious	Probably damaging	Probably damaging	High	Neutral	Disease	Deleterious	Effect	Deleterious	Neutral	Disease	Deleterious
rs79901429	I204T	Deleterious	Probably benign	Probably damaging	Medium	Disease	Disease	Deleterious	Effect	Deleterious	Neutral	Disease	Deleterious
rs761626260	L207W	Deleterious	Probably damaging	Probably damaging	Medium	Disease	Neutral	Deleterious	Effect	Deleterious	Disease	Disease	Neutral
rs377085266	C212R	Deleterious	Probably damaging	Probably damaging	Medium	Disease	Disease	Deleterious	Effect	Deleterious	Disease	Disease	Neutral
rs780065109	G231V	Deleterious	Probably damaging	Probably damaging	Medium	Disease	Disease	Deleterious	Effect	Deleterious	Disease	Disease	Deleterious
rs781105138	L235P	Deleterious	Probably benign	Probably damaging	Medium	Disease	Disease	Deleterious	Effect	Deleterious	Disease	Disease	Deleterious
rs1142345	Y240C	Deleterious	Probably damaging	Probably damaging	High	Disease	Disease	Deleterious	Effect	Deleterious	Neutral	Disease	Deleterious
rs1142345	Y240S	Deleterious	Probably damaging	Probably damaging	Medium	Neutral	Disease	Deleterious	Neutral	Deleterious	Disease	Disease	Deleterious

**Table 2:** List of missense nsSNPs predicted to be deleterious by at least six out of eight structure-based tools (As Y240S has already been identified to be deleterious, it is not a novel deleterious SNP and hence, it was not considered for further in silico analyses).

Variant ID	SNP	CUPSAT	DUET			I-Mutant	MUpro		INPS-MD	PoPMuSiC	HoTMuSiC	SNPMuSiC
			mCSM	SDM	DUET		SVM*	NN*				
rs72552741	W33G	Destabilising	Destabilizing	Destabilizing	Destabilizing	Decrease	Decrease	Decrease	Decrease	Strongly Decrease	Strongly Decrease	Strongly Decrease
rs753277177	W78R	Destabilising	Destabilizing	Destabilizing	Destabilizing	Decrease	Decrease	Decrease	Decrease	Decrease	Decrease	Strongly Decrease
rs1784191846	V89E	Destabilising	Destabilizing	Destabilizing	Destabilizing	Decrease	Decrease	Decrease	Decrease	Strongly Decrease	Strongly Decrease	Decrease
rs1447033392	W150G	Destabilising	Destabilizing	Destabilizing	Destabilizing	Decrease	Decrease	Decrease	Decrease	Strongly Decrease	Strongly Decrease	Strongly Decrease
rs1386533390	L182P	Destabilising	Destabilizing	Destabilizing	Destabilizing	Decrease	Decrease	Decrease	Decrease	Strongly Decrease	Strongly Decrease	Decrease
rs1142345	Y240S	Destabilising	Destabilizing	Destabilizing	Destabilizing	Decrease	Decrease	Decrease	Decrease	Strongly Decrease	Decrease	Decrease

*SVM = Support Vector Machines Method, NN = Neural Networks Method.

### Validation of the screening pipeline

It is a well-known fact that in silico predictions and analyses are not 100% accurate and are not as conclusive as in vitro or in vivo experiments. However, that does not mean that in silico predictions should be completely disregarded. In order to validate the in silico methodology utilized in this study, other human TPMT missense SNPs already identified as deleterious through experimental means were subjected to the same screening pipeline, with a minor difference as highlighted in “Validation of the screening pipeline” section. The results of the screening process on missense SNPs of TPMT, pre-identified as deleterious experimentally in other studies^9,11,15,20,72–92^ are summarised in Table 3 and are presented more comprehensively in Table S3.

**Table 3:** Screening of pre-identified SNPs to validate the screening process (SNPs that would get selected as per the screening process utilised in this study are highlighted in bold. PV = PROVEAN, PT = PANTHER, PP2 = PolyPhen-2, MA = Mutation Assessor, MS = Meta-SNP, PM = PMut, PS1 = PredictSNP1, S2 = SNAP 2, SP = SuSPect, SG = SNPs&GO, PS = PhD-SNP, ST = SIFT, C = CUPSAT, D = DUET, IM = I-Mutant, MP = MUpro, IMD = INPS-MD, PPM = PoPMuSiC, HTM = HoTMuSiC, SM = SNPMuSiC, VD = Very Dangerous, D = Dangerous, N = Neutral).

Variant ID	SNP	Sequence-based tools (12)												Structure-based tools (8)								Tools (20)	Nature
		PV	PT	PP2	MA	MS	PM	PS1	S2	SP	SG	PS	ST	C	D	IM	MP	IMD	PPM	HTM	**SM**		
rs9333569	M1V	N	D	N	NA*	N	N	N	N	N	N	N	D	NA*	NA*	NA*	NA*	NA*	NA*	NA*	NA*	NA*	D ^84^ ,VD ^77^
rs72552742	E28V	D	N	N	N	N	D	N	D	N	N	D	D	D	N	N	D	N	D	D	N	9	D ^77,88,91^
NA**	Q42E	N	N	N	N	N	N	N	N	N	N	N	N	D	N	N	N	N	D	D	N	3	D ^77,81^
**rs72552740**	**L49S**	**D**	**D**	**D**	**D**	**D**	**D**	**D**	**D**	**D**	**D**	**D**	**D**	**D**	**D**	**D**	**D**	**D**	**D**	**D**	**D**	**20**	**VD** ^77,85,88^
**rs200591577**	**L69V**	**D**	**D**	**D**	**D**	**D**	**D**	**D**	**D**	**D**	**D**	**D**	**D**	**D**	**N**	**D**	**D**	**D**	**D**	**D**	**N**	**18**	**VD** ^75,83^
**rs777686348**	**G71R**	**D**	**D**	**D**	**D**	**D**	**D**	**D**	**D**	**D**	**D**	**D**	**D**	**D**	**D**	**D**	**D**	**N**	**D**	**D**	**D**	**19**	**VD** ^77,81^
rs281874771	A73V	D	D	D	N	D	D	D	D	D	N	N	D	D	D	N	N	N	D	D	D	14	VD ^89^
**rs1800462**	**A80P**	**D**	**D**	**D**	**D**	**D**	**D**	**D**	**D**	**D**	**D**	**D**	**D**	**D**	**D**	**D**	**D**	**D**	**D**	**D**	**D**	**20**	**VD** ^77,78,87^
rs111901354	R82W	D	N	D	N	D	N	D	D	D	D	D	D	D	D	D	N	N	D	D	N	14	VD ^74^
NA**	**Y107D**	**D**	**D**	**D**	**N**	**D**	**D**	**D**	**D**	**N**	**D**	**D**	**D**	**D**	**D**	**D**	**D**	**D**	**D**	**D**	**D**	**18**	**VD** ^76^
rs115106679	E114K	N	N	D	N	N	N	N	N	N	N	N	D	D	N	D	D	N	D	D	N	7	VD ^74,86^
rs200220210	S125L	D	N	N	N	N	N	N	N	N	N	N	N	D	N	N	N	N	D	D	D	5	D ^77,88,91^
**rs72552738**	**C132Y**	**D**	**D**	**N**	**N**	**D**	**D**	**D**	**N**	**D**	**D**	**D**	**D**	**N**	**D**	**D**	**D**	**D**	**D**	**D**	**D**	**16**	**D** ^77,91^**, VD** ^80^
**rs72552737**	**G144R**	**D**	**D**	**D**	**N**	**D**	**N**	**D**	**D**	**N**	**D**	**D**	**D**	**D**	**D**	**D**	**D**	**N**	**D**	**D**	**D**	**16**	**D** ^77,88,91^**, VD** ^15^
**rs1800460**	**A154T**	**D**	**N**	**N**	**D**	**D**	**D**	**D**	**D**	**D**	**D**	**D**	**D**	**N**	**D**	**D**	**D**	**N**	**D**	**D**	**D**	**16**	**VD** ^11,73,78,82,87,89^
**rs112339338**	**R163C**	**D**	**D**	**D**	**D**	**D**	**D**	**D**	**D**	**D**	**D**	**D**	**D**	**N**	**D**	**D**	**D**	**N**	**D**	**D**	**D**	**18**	**VD** ^74^
rs144041067	R163H	D	D	D	N	D	N	D	D	D	N	D	N	D	D	D	D	D	D	D	N	15	VD ^72,77,81^
NA**	**R163P**	**D**	**D**	**D**	**D**	**D**	**D**	**D**	**D**	**D**	**D**	**D**	**D**	**N**	**D**	**D**	**D**	**D**	**D**	**D**	**D**	**19**	**VD** ^75,77^
**rs201695576**	**Y166C**	**D**	**D**	**D**	**D**	**D**	**D**	**D**	**D**	**D**	**D**	**D**	**D**	**N**	**D**	**D**	**D**	**D**	**D**	**D**	**D**	**19**	**D** ^79^
rs74423290	A167G	D	N	N	N	N	D	D	D	D	N	D	N	D	D	D	D	D	D	D	D	15	D ^92^
rs72556347	F208L	N	D	N	N	N	N	N	N	N	N	D	N	D	D	D	D	D	D	D	D	10	D ^90^
**rs377085266**	**C212R**	**D**	**D**	**D**	**N**	**D**	**D**	**D**	**D**	**D**	**D**	**D**	**N**	**D**	**D**	**D**	**D**	**D**	**D**	**D**	**D**	**18**	**VD** ^83^
rs150900439	K238E	N	N	N	N	N	N	N	N	N	N	N	D	N	N	D	D	N	D	D	N	5	D ^75,77^
**rs1142345**	**Y240C**	**D**	**D**	**D**	**D**	**D**	**D**	**D**	**D**	**D**	**N**	**D**	**D**	**D**	**D**	**D**	**D**	**N**	**D**	**D**	**D**	**19**	**VD** ^9,11,73,78,82,87^
**rs1142345**	**Y240S**	**D**	**D**	**D**	**N**	**N**	**D**	**D**	**N**	**D**	**D**	**D**	**D**	**D**	**D**	**D**	**D**	**D**	**SD**	**D**	**D**	**18**	**VD** ^20^

*NA = Not Available (Residues 1 to 16 are absent in the PDB file 2H11, hence structural analysis tools could not be used for this SNP. Mutation Assessor also did not yield any result for this SNP).

**NA = Not Available (This pre-identified SNP is not annotated in dbSNP).

**Figure 2:**
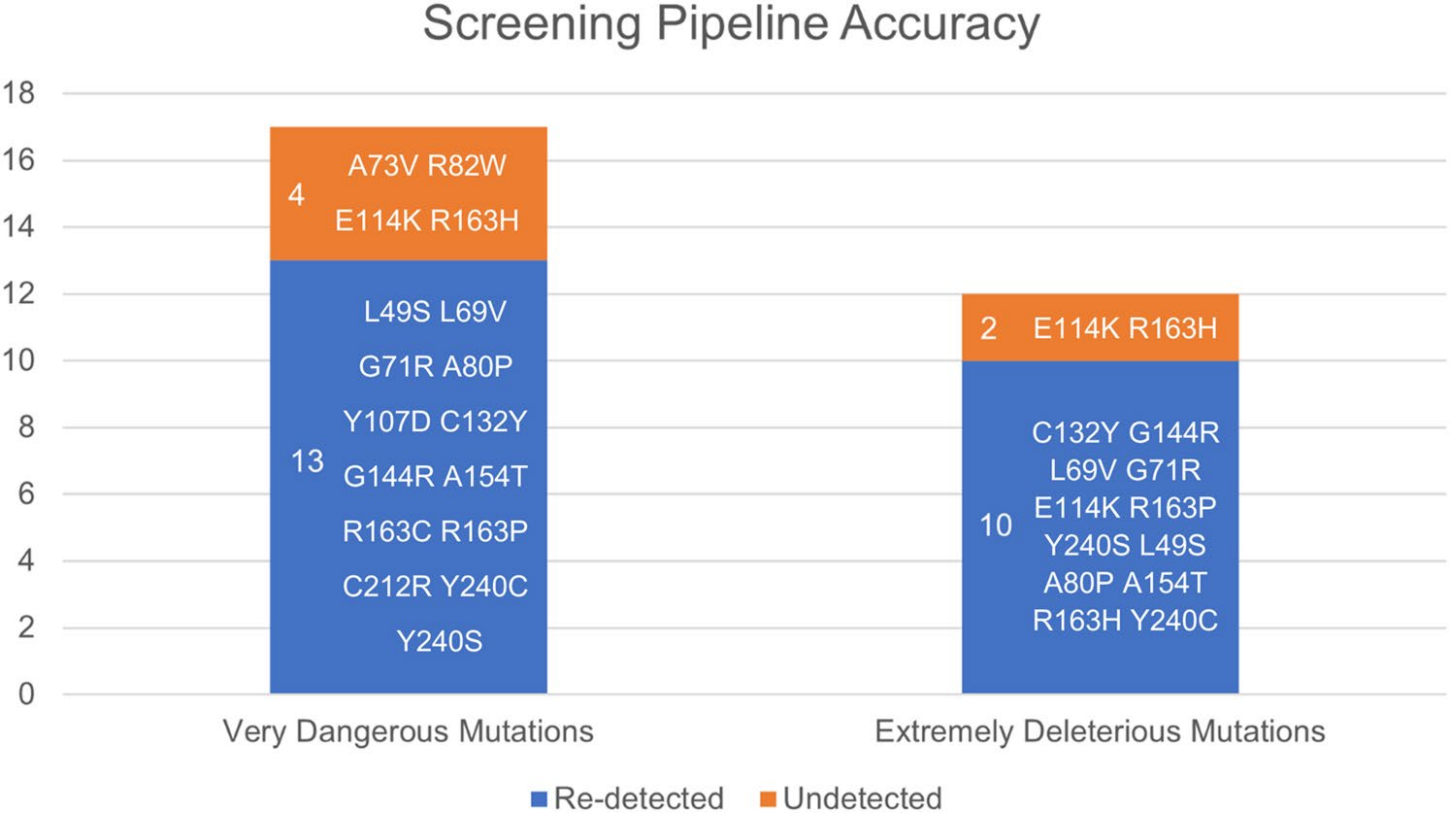
Accuracy of the in silico screening pipeline with respect to the pre-identified deleterious missense mutations of human TPMT.

13 out of the 17 (76.47%) very dangerous mutations, excluding M1V, were re-detected using the screening pipeline. M1V was not considered as that residue is not present in the structure of TPMT (2H11) and hence, structure-based screening could not be performed for M1V. Furthermore, with respect to extremely deleterious mutations which were proven to be very dangerous by one or more studies and/or dangerous by multiple studies, 10 out of 12 (83.33%) of them were re-detected. Figure 2 visually represents the accuracy of the screening pipeline.

ConSurf, which was used for evolutionary conservation analysis, predicts colour scores for residues that can range between 1 and 9 and are a measure of the conservation level of that particular residue. A residue can be considered as extremely variable if it has a score of “1” and conversely, it can be considered as extremely conserved if it has a score of “9”. It was observed that while high conservation of the residues which are predicted to harbor SNPs provides further validation of their deleterious nature, it is not completely conclusive. This is due to the fact that in the case of five SNPs (C132Y, G144R, C212R, Y240C and Y240S) pre-identified as extremely deleterious and also redetected by the screening pipeline, the color scores predicted by ConSurf were lower than or equal to 5. However, nine SNPs pre-identified as extremely dangerous (L49S, L69V, G71R, A80P, Y107D, A154T, R163C, R163P and Y166C) and also re-detected using the screening pipeline did have color scores predicted as greater than or equal to 6. Hence, while conservation analysis is good as a secondary form of validation of the deleterious nature of screened SNPs, it is not guaranteed that if an SNP is at a relatively less conserved residue it will be benign in nature. In fact, in a similar study on the GOT1 gene, an SNP (L345P) was predicted to be highly deleterious despite having a color score (predicted by ConSurf) of 1 and its deleterious nature was reflected in further in silico analyses of that study^93^.

### Conservation analysis

The level of conservation of the amino acid residues provides a rough estimate regarding the level of damage that dangerous mutations can cause to the functioning of the protein and its structure. As mentioned before, they are not completely conclusive but do provide a good form of secondary validation of the deleterious nature of the SNP in question. The Bayesian method was used to obtain the ConSurf results, and the protein structure (2H11) was given as the input. 10 sequences of TPMT including human TPMT (P51580) were considered for MSA, and scores ranging from 1 to 10 were assigned based on how many sequences had the same amino acid residue at the residue location under consideration. For instance, the amino acid “W” or tryptophan was conserved at residue 33 across all 10 sequences and hence, was assigned a score of 10 for MSA. If an amino acid was at a particular residue location only for human TPMT and none of the other nine TPMT sequences, it would be assigned a score of 1. As for CDD, 13 of the most dissimilar sequences of TPMT were considered for MSA, and scores ranging from 1 to 13 were considered, assigned similarly to MSA depending on how many sequences had that particular amino acid conserved at the given residue location.

The ConSurf results showed that four of the five screened mutations, W33G, W78R, V89, and W150G were present at residues that were highly conserved as all of them were on residues possessing a color score of 8 or 9. Residue 182 (where the L182P mutation occurs) had a slightly above-average conservation score of 6. The color scores corresponding to all amino acid residues of TPMT as predicted by ConSurf are shown in Table S4. They are structurally represented for chain A of TPMT (2H11) in Figure S2 as a still image and as an animation for better visualization through the POLYVIEW-3D server^94^ in Animation 1. The conservation analysis results obtained through the five MSA tools, ConSurf and CDD with respect to the residues associated with the five screened SNPs are presented in Table S5. All of them were conserved across the 10 aforementioned species whose TPMT sequences were considered for MSA. In addition, CDD which identified the most diverse TPMT sequences and performed MSA found that all but one SNP (W150G) were at residues that were conserved across at least seven of the most dissimilar TPMT sequences indicating that those residues have stood the test of time and evolution to remain a part of the amino acid sequence of the protein, presumably due to their important contribution to the desired functioning or maintenance of structural stability of the protein. It is worth noting that this does not invalidate the candidature of W150G as a potentially deleterious missense SNP of TPMT, since despite the CDD results, residue 150 had a color score of 8 as per ConSurf results. Furthermore, conservation level is not an absolute indicator, but rather a good general indicator of deleterious nature.

As per ConSurf, 108 residues (46.55%) possessed color scores that were greater than or equal to 6, and 47 (18.1%) among them were highly conserved having a conservation score of 8 or 9. Among these 108 residues, the largest conserved region consisted of residues 64–110 (except residues 85, 91, 94, 97, 98, 101, 104, 106, and 108) having 38 residues with color scores greater than or equal to 6 and 20 highly conserved residues having color scores greater than or equal to 8. The regions covering the ligand (S-adenosyl-L-methionine or SAM) attachment site are from residues 29 to 40 and 134 to 135. Hence, ligand binding does not explain the high conservation level of this region. Further investigation is required to deduce the high level of conservation observed in this region.

### Interaction analysis

The Search Tool for the Retrieval of Interacting Genes/Proteins (STRING) database was used to identify the interaction network of human TPMT. The output was given in the form of edges and nodes that represent interactions and proteins, respectively, as shown in Figure S3. All of the predicted functional partners of TPMT and their respective scores are shown in Table S6. These scores indicate how likely an interaction is to be true, as evaluated by STRING.

The interaction analysis revealed that the TPMT interaction network is primarily involved in the biosynthesis of guanosine monophosphate (GMP) and guanosine triphosphate (GTP), catabolism of deoxyribonucleoside triphosphate (dNTPs) and the biosynthesis as well as degradation of purines. GMP is a nucleotide that is utilized as a monomer in RNA. GTP is an extremely essential nucleotide in the body serving as a medium of energy transfer in the cell, similar to adenosine triphosphate (ATP), albeit more specific than its more universal counterpart, ATP. It is also important in signal transduction, most prominently in G-proteins. Furthermore, cyclic GMP (cGMP) which is derived from GTP, is a very important second messenger in the body involved in key biological processes, including but not limited to glycogenolysis, cellular apoptosis, vasodilation, phototransduction in the eye, and more. Due to the importance of the network with regard to purines and dNTPs, it is very important for the regular functioning of most cellular processes. Interestingly, the network is also involved in lymphocyte proliferation reaffirming an immune response association which is in stark contrast to the importance of TPMT in the methylation of thiopurine drugs that treat autoimmune disorders like Crohn’s disease and rheumatoid arthritis.

Suffice it to say that the TPMT interaction network is a very important network involved in several key biological processes. All major biological processes in which the TPMT interaction network is involved, as per the STRING database are shown in Table S7. As mentioned before, TPMT polymorphisms can severely alter enzyme activity and the appropriate conduction of these key biological processes that the network is involved in, are subject to the deleterious consequences of these dangerous polymorphisms.

### Oncogenic analysis and phenotypic analysis

As mentioned before, TPMT polymorphisms are known to be associated with cancers. Hence, to predict the oncogenic nature of the screened SNPs, CScape and CScape-somatic were used. The former predicts the oncogenic nature of somatic point mutations that occur in the coding regions of the cancer genome with an accuracy of 92%. The input is given using the format**:** chromosome, position, reference, mutant; as per the GRCh38 assembly for one or multiple mutations. The output is in the form of p-values which can lie between 0 and 1 and values above 0.5 are considered deleterious. On the other hand, values below 0.5 are considered benign. It predicted that all five mutations are oncogenic and W33G with high confidence. CScape-somatic is a tool that can differentiate between mutations that can be cancer drivers and mutations that can be passenger variants. Cancer drivers occur fairly early in the development of the tumor. Meanwhile, passenger variants accumulate at later stages after a tumor starts to grow and usually correspond to low or no oncogenicity. The input and accuracy of CScape-somatic are identical to CScape, except for the fact that the GRCh37 assembly is used in the input. Akin to CScape, the predictions of CScape-somatic are again given as p-scores that lie between 0 and 1. Mutations that possess p-scores above 0.5 are predicted to be cancer drivers. Conversely, mutations that possess p-scores 0.5 are predicted to be passenger variants. CScape-somatic predicted that all five mutants are cancer drivers and W33G with high confidence. The results obtained from both oncogenic tools are presented in Table 4.

**Table 4:** **:** Oncogenic nature of mutations predicted using CScape and CScape-somatic.

Variant ID	SNP	CScape			CScape-somatic		
		**Input & Assembly**	**Coding Score**	**Message**	**Input & Assembly**	**Coding Score**	**Message**
rs72552741	W33G	6,18,149,031,A,C & GRCh38	0.898663	Oncogenic (HC*)	6,18,149,262,A,C & GRCh37	0.907987	Driver (HC*)
rs753277177	W78R	6,18,147,824,A,G & GRCh38	0.695307	Oncogenic	6,18,148,055,A,G & GRCh37	0.820811	Driver
rs1784191846	V89E	6,18,143,696,A,T & GRCh38	0.807748	Oncogenic	6,18,143,927,A,T & GRCh37	0.781448	Driver
rs1447033392	W150G	6,18,139,009,A,C & GRCh38	0.860191	Oncogenic	6,18,139,240,A,C & GRCh37	0.728649	Driver
rs1386533390	L182P	6,18,133,839,A,G & GRCh38	0.519117	Oncogenic	6,18,134,070,A,G & GRCh37	0.841487	Driver

*HC = High Confidence.

In addition to CScape and CScape-somatic, Dr Cancer was also used for the assessment of the oncogenic potential of SNPs. It is a slightly older tool than the CScape tools that utilize a disease-specific machine learning approach to predict whether a missense SNP is associated with cancer or not. It does so by utilizing multiple methods including SEQPROF where the sequence and profile at the mutated position are the input for the Support Vector Machine (SVM), SVM-GOS where the GOS (Gene Ontology Score) of the mutated sequence is the input for the SVM, and finally, SEQPRFGO where the input for the SVM is the input provided for the two prior methods. The first method, SEQPROF predicted that all five mutations are cancer-associated while the other two methods did not predict any of the mutations to be cancer-associated. The results pertaining to the five mutations obtained from Dr Cancer are shown in Table S8.

Functional Analysis through Hidden Markov Models (FATHMM) server was used for phenotypic analysis as it predicts the phenotypic consequences of mutations in the human genome. The input given was the Uniprot ID of TPMT along with the amino acid substitutions associated with the five deleterious missense SNPs. The tool classified only L182P as a dangerous mutation, associated with abnormalities of the head, neck, face, mouth, and philtrum. The results obtained from the FATHMM server are presented in Table S9.

### Structural effects of deleterious missense SNPs

W33G at residue 33 is present at a ligand binding site and will undoubtedly have an effect on the ligand binding process and subsequent functioning of the protein. V89E at residue 89 and W150G at residue 150 is extremely close to the ligand binding sites at residues 90 and 150 respectively and hence, they may also impact the ligand binding process. The HOPE server was used to visualize the five highly deleterious missense SNPs, as shown in Animation 2. It was found that except V89E, all mutant residues were smaller in size when compared to their wild-type counterparts. Hence, with respect to W33G, W78R, W150G, and L182P, it was predicted that the mutant residues would not fit in the core of the protein since they are larger than the respective wild-type counterparts. All structural consequences of the mutations as predicted by the HOPE server have been presented in Table S10.

The secondary structure alterations caused by the mutations, as obtained from NetSurfP-2.0 are shown in Figure S4. It was observed that L182P caused the helix to start from residue 47 instead of residue 46 in the wild-type structure of TPMT (2H11). However, NetSurfP-2.0 predicted this coil to start at residue 47 itself, which is incorrect. Regardless, if the predicted change does occur, it might be damaging due to the change induced with respect to the helix. W33G and L182P replaced a coil with a strand at residue 122. However, in the wild-type structure of TPMT (2H11), this residue is already under the conformation of a strand. This is because NetSurfP-2.0 did not correctly predict the confirmation at residue 122 for the wild-type sequence of TPMT. Since the wild-type structure of TPMT already has that conformation at residue 122, if the mutation causes the predicted change, it will make no difference. A similar change was observed with respect to W33G and V89E, but here the strand was replaced by a coil at residue 68. However, the wild-type structure of TPMT (2H11) already has a coil at residue 68 which was not predicted by NetSurfP-2.0. Hence, this predicted change will also cause no damage. Yet another change akin to the aforementioned changes was observed where L182P replaced a strand with a coil at residue 187, but in the wild-type structure of TPMT, this residue is already in the confirmation of a coil. Hence, this predicted change will also cause no damage. Due to the inconsistencies between the secondary structure prediction of wild-type TPMT by NetSurfP-2.0 and the actual wild-type structure of TPMT, it is difficult to draw any concrete conclusions regarding the secondary structure changes. Hence to infer some credible changes caused by the mutations in the secondary structure and other structural aspects of the protein, MDS was carried out for a period of 100 ns, the results of which are discussed in “Molecular dynamics simulations analysis” section.

The CASTp 3.0 server was used to assess the alterations in the binding pockets caused by the mutations. The results for the same are shown in Table S11. It was observed that W33G and W150G vastly increased the size of the largest binding pocket of TPMT which will undoubtedly impact ligand binding as residue 33 is a ligand binding site. W78R and V89E did not impact the binding pocket dimensions significantly. L182P introduced a few pockets that are absent from the wild-type protein albeit of a very minuscule size that is unlikely to impact the binding process much. However, it also introduced a larger binding pocket that is absent from the wild-type protein. This might affect the binding process.

Finally, AmylPred 2 was used to identify amyloid-forming regions in TPMT. Amyloid refers to proteinaceous and abnormally fibrous extracellular deposits. It is insoluble, mostly possesses a β-sheet conformation and is associated with a variety of diseases called amyloidoses, such as Alzheimer's disease, type 2 diabetes and more. They are usually progressive disorders associated with high morbidity and mortality^95^. The amyloid-forming regions predicted by AmylPred 2 for TPMT include residue groups 64–69, 86–89, 100–101, 127–138, 147–149, 154–158, 170–175, 178–187, 207–217 and 231–243. W150G is just one residue away from the predicted amyloid region comprised of residues 147–149, while V89E and L182P fall under these regions with residues 86–89 and 178–187, respectively. Hence, they may influence these predicted amyloid- forming regions and worsen disease progression.

### Post-translational modification analysis

GPS 5.0 software was used to predict the PTM sites of TPMT. The predicted PTM sites and their distribution are shown in Figure S5. While none of the mutations are at PTM sites, W33G is near the PTM site T38, V89E is in proximity to S92 and S85. Furthermore, L182P is in close proximity to the PTM site Y180. It is very much possible that due to their proximity to PTM sites, W33G, V89E and L182P can impact the phosphorylation of the protein, which is essential for the natural functioning of the protein.

### Molecular dynamics simulations analysis

MDS was performed to identify the structural consequences of the mutations in a dynamic fashion and at an atomic level. Seven systems (Wild-Type Apo protein (WT), Wild-Type protein with SAH ligand attached (WT-SAH), W33G, W78R, V89E, W150G, and L182P) were generated for MDS, following which MDS was performed for a simulation time of 100 ns through GROMACS. All of the 10,000 frames from the 100 ns of simulation time were recorded for the WT, WT-SAH, W33G, W78R, V89E, W150G, and L182P systems and are shown in Animations 3, 4, 5, 6, 7, 8, and 9 respectively. From Animation 4 (WT-SAH) and Animation 5 (W33G) it can be clearly seen that the ligand interactions upon stabilization do not resemble each other. As mentioned before, W33G occurs at a ligand binding site and is undoubtedly the reason for the difference in ligand interactions between the W33G mutant and the wild-type protein. From Animation 6 (W78R) it is apparent that the ligand interactions are different from the wild-type protein, however not to the extent observed in W33G. The difference is even less apparent in V89E (Animation 7). However, from Animation 8 (W150G), it can be clearly seen that the ligand interactions in the W150G mutant, upon stabilization, are very different from the wild-type protein. Furthermore, from Animation 9 (L182P), it can also be seen that the ligand interactions are different from the wild-type protein. These results are in line with the CASTp 3.0 results where W33G and W150G affected the largest binding pocket of TPMT and L182P introduced and altered several pockets, while W78R and V89E did not have much impact on the binding pockets. Trajectory analysis and MM-PBSA analysis were performed in order to quantify the MDS results.

### RMSD analysis

**Figure 3:**
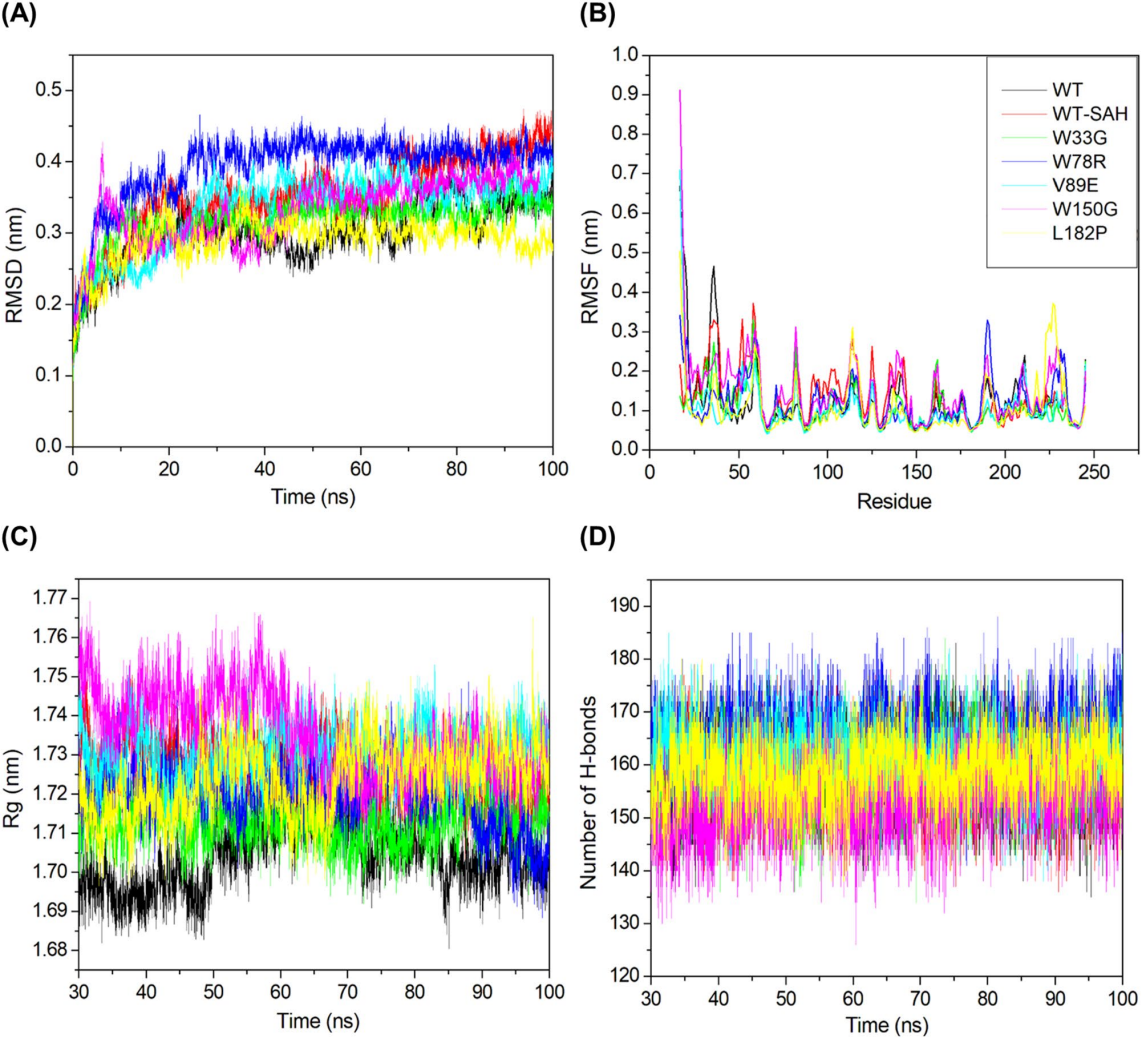
RMSD, RMSF, Rg and H-bond analysis results.

RMSD was calculated for all seven protein systems to assess their respective stabilities. RMSD as a parameter is useful for assessing the stability of the protein relative to its conformation. Smaller RMSD deviations correspond to a more stable protein structure. A plot of RMSD against time for all systems is shown in Fig. 3A. The apo form of the wild-type protein and the ligand-bound form had average RMSD values of 0.301 nm and 0.353 nm, respectively. From Fig. 3A, it can be seen that the apo form of TPMT is more stable than the ligand-bound TPMT and the average RMSD values reflect the same. Meanwhile, the mutants W33G, W78R, V89E, W150G and L182P had average RMSD values of 0.317, 0.392, 0.335, 0.331 and 0.291 nm, respectively. It was also observed that the W150G mutant showed the most RMSD fluctuations till the first 50 ns. Interestingly, the L182P mutant protein appeared to possess higher stability than both the apo form and ligand-bound wild-type conformations of the protein. Furthermore, only W78R had an average RMSD value higher than ligand-bound TPMT, implying all other mutations are more stable than ligand-bound TPMT as per RMSD analysis. The RMSD results indicated that all systems attained stability after 30 ns. Hence, the last 70 ns of the trajectory were considered with respect to the calculations pertaining to the rest of the MDS parameters.

### RMSF analysis

RMSF was used to assess the flexibility and stability of the seven systems. High RMSF values usually signify greater flexibility during MDS. A plot showcasing the RMSF values against all residues for all systems is shown in Fig. 3B. The apo form of the wild-type protein and the ligand-bound form had average RMSF values of 0.120 nm and 0.132 nm, respectively, again indicating that the apo form of TPMT is more stable than the ligand-bound TPMT. Meanwhile, the mutants W33G, W78R, V89E, W150G, and L182P had average RMSF values of 0.108, 0.123, 0.100, 0.148, and 0.108 nm, respectively. Interestingly, mutants W33G, V89E, and L182P appear to be more stable than both the apo form and ligand-bound wild-type conformations of the protein. Furthermore, only W150G had an average RMSF higher than ligand-bound TPMT, implying all other mutations are more stable than ligand-bound TPMT at least in the 100 ns of simulation time and as per RMSF.

### Radius of gyration analysis

The radius of gyration (Rg) of a system indicates the root-mean-square distance of the atoms of the protein in the system from the axis of rotation and is useful to assess protein structure compactness. Low Rg values indicate a more compact and rigid protein structure, while high Rg values indicate that the protein is less compact and flexible. A plot showcasing the variation of Rg with respect to time for all systems is shown in Fig. 3C. The apo form of the wild-type protein and the ligand-bound form had average Rg values of 1.704 nm and 1.726 nm, respectively. Hence, the apo form of TPMT is less flexible than the ligand-bound form of TPMT. Meanwhile, the mutants W33G, W78R, V89E, W150G, and L182P had average RMSF values of 1.713, 1.721, 1.730, 1.735, and 1.724 nm, respectively. The apo form of the protein was found to be the most stable among all systems. V89E and W150G were the only systems that were less compact and more flexible than ligand-bound TPMT, implying all other mutations are more stable as per the Rg values.

### Hydrogen bonding analysis

Hydrogen bonds are important for maintaining the stability of the protein structure. A greater number of average hydrogen bonds correspond to a more stable protein structure, and conversely, a fewer number of average hydrogen bonds correspond to a less stable protein structure. A plot showcasing the number of intramolecular hydrogen bonds against time for all systems is shown in Fig. 3D. The apo form of the wild-type protein and the ligand-bound form had 158 and 157 hydrogen bonds on average, respectively. While this does mean that, yet again ligand-bound TPMT is less stable than the apo form of TPMT, the difference is very minuscule. Meanwhile, the mutants W33G, W78R, V89E, W150G, and L182P had 158, 165, 160, 153, and 159 hydrogen bonds on average, respectively. W33G and L182P seemed to have a negligible effect on the hydrogen bond formation. However, apart from W150G, the average number of hydrogen bonds was more than or equal to the average hydrogen bonds in the apo form of TPMT, implying that all other mutations are more stable than the apo form of TPMT.

### SASA analysis

**Figure 4:**
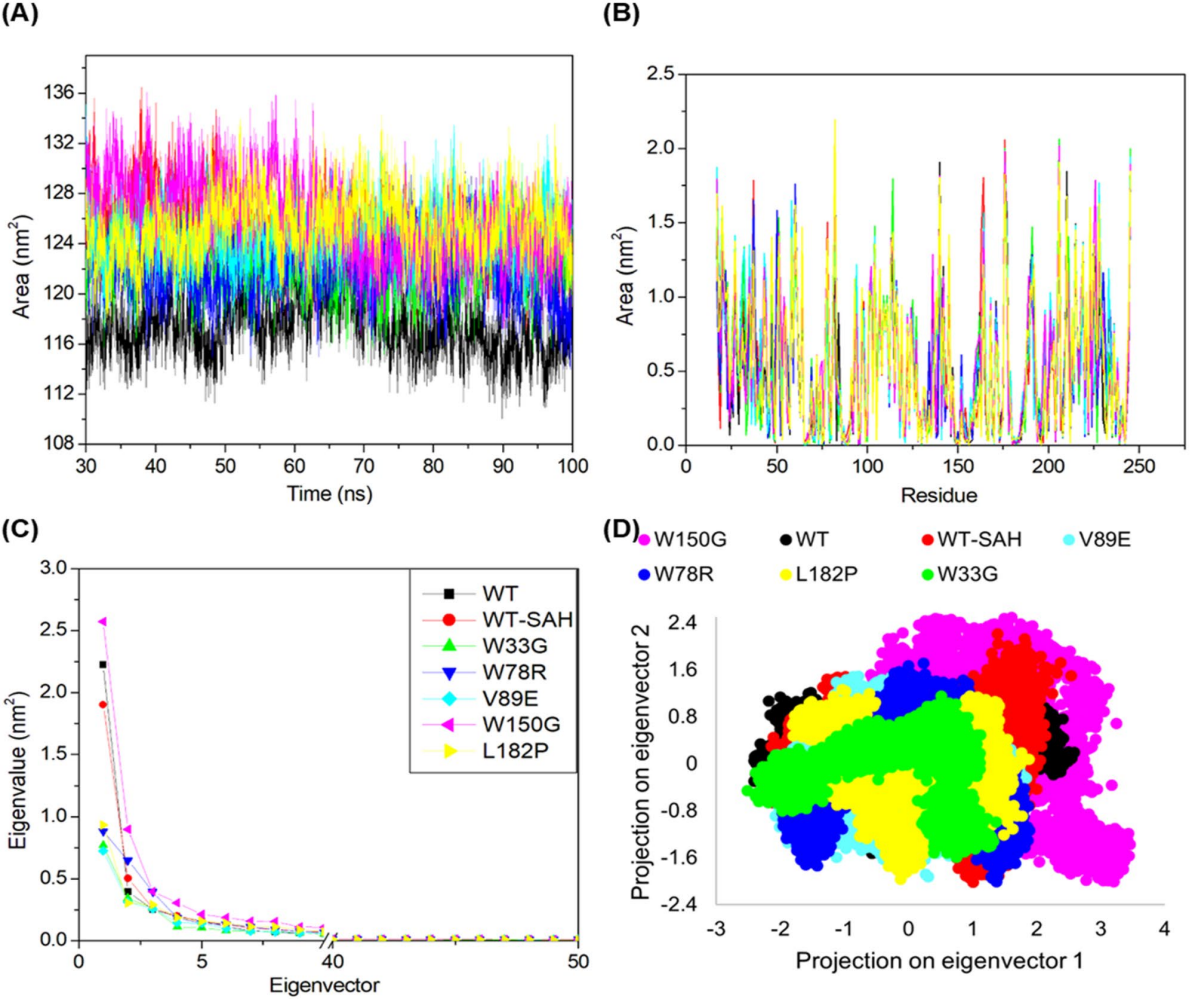
SASA analysis and PCA results.

SASA as a parameter is useful for assessing the portion of the protein surface that is exposed to the water solvent. A plot of SASA against time for all systems is shown in Fig. 4A, while a plot of SASA against all residues for all systems is shown in Fig. 4B. The apo form of the wild-type protein and the ligand-bound form had average SASA values of 117.414 nm^2^ and 124.768 nm^2^, respectively. Their average residual SASA values were found to be 0.512 nm^2^ and 0.545 nm^2^, respectively. Both parameters indicate that the ligand-bound TPMT has a hydrophobic core that is more exposed to the solvent than the apo form of TPMT than the apo form of TPMT. This could be essential for the appropriate functioning of the protein. Meanwhile, the mutants W33G, W78R, V89E, W150G, and L182P had average SASA values of 122.214, 121.862, 124.490, 126.017, and 125.385 nm^2^, respectively, and average residual SASA values of 0.534, 0.532, 0.543, 0.550 and 0.557 nm^2^, respectively. Hence, mutants W33G, W78R, and V89E have more hydrophobic cores that are more protected from the solvent when compared to the ligand-bound form of TPMT. However, this could be detrimental as a certain degree of flexibility is important for the normal functioning of the protein. On the other hand, mutants W150G and L182P appeared to have more hydrophobic cores that are more exposed to the solvent than ligand-bound TPMT as per the SASA analysis.

This is in agreement with respect to the results from prior analyses where the apo form of TPMT was always found to possess more stability than ligand-bound TPMT, however excess stability or plasticity of the protein can actually hamper its natural functioning and a certain degree of flexibility is needed for the protein to bind to the ligand and perform its intended function^96^. From the results obtained thus far, it would seem that most mutations increase the plasticity of the protein structure and thereby reduce its functional capabilities. In fact, in another study where MDS was performed for the Y240S mutant of TPMT, the RMSD graph of the Y204S mutant showed a lesser average value than wild-type TPMT, despite the fact that in the same study Y204S was proven to be very deleterious through in vivo techniques^19^. Another study found higher SASA values in the three most well-known and deleterious TPMT alleles (A80P, A154T, and Y240C) than the SASA values of WT-TPMT^97^. Hence, it is not unprecedented for enhanced stability resulting from missense mutations to have a negative effect on TPMT. Hence, these results are in line with prior mutational studies of TPMT. To further validate this phenomenon of functional hampering due to enhanced plasticity in the case of TPMT polymorphisms, the MM-PBSA method was utilized to assess the binding energy and its components for all six ligand-bound systems in “Binding energy analysis” section.

### PCA

PCA was also performed in order to assess the collective motions of the seven systems. The motions of the Cα atoms of a given protein were the basis of PCA. Eigenvalues were used to quantify the atomic contribution to the collective motion and eigenvectors were used to quantify the overall direction of motion of atoms. The eigenvalues were plotted in decreasing order against the respective eigenvector for all seven systems in Fig. 4C. As is evident from Fig. 4C, apart from W150G, the eigenvalues of all mutations possessed amplitudes lower than that of the apo form and the ligand-bound form of TPMT, implying more rigidity in the collective motions upon mutations. This is in line with prior analyses as W150G was the only mutation that was predicted to be unstable by three parameters (RMSF, Rg, H-bonds, SASA). The first two eigenvectors were plotted oppositely in phase space where each of the continuum spectra represents the correlated motions. The analyzed clusters of all seven systems were plotted and are depicted in Fig. 4D. As is evident from prior analyses, most mutations with the exception of W150G, would be expected to reduce the collective motion of the system due to the enhanced plasticity. The PCA results line up with this expectation as it can be seen in Fig. 4D that apart from W150G, the collective motions of all mutations are more restricted than both the apo form and the ligand-bound form of TPMT, with W33G having the most restricted motion. This lines up with the Rg results where W33G was found to be the most rigid mutation system. Hence, it was observed that none of the mutant systems developed motion clusters that were similar to that of the apo form or ligand-bound form of TPMT, reaffirming the fact that they have a deleterious impact on TPMT.

### Secondary structure analysis

SSA was performed for all systems, and the results for the same are presented in Figure S6. In the apo form of the protein, a 5-turn helix conformation exists between residues 17 and 27 after 85 ns. However, in the WT-SAH system, the residues were not in this conformation after 85 ns. Furthermore, close to residue 177, WT-SAH replaced the β-sheet conformation observed in the apo form, with bends. It also replaced the α-helix near residue 137 with turns for a brief period but stabilized similarly to the apo form near the end of simulation time. Most notably, the α-helix between residues 222 and 237 was completely replaced by bends in the WT-SAH system over the entire simulation time.

All mutant comparisons regarding secondary structure are with respect to the WT-SAH system, as all mutant systems are also ligand-bound. It was observed that in all mutant systems, residues 17 to 27 seemed to lose a lot of their secondary structure conformations, which is problematic as residue 26 is a ligand binding site. With respect to the W33G system, it very notably replaced the α-helix conformation between residues 27 and 37 with a 5-turn helix conformation for nearly the entirety of the simulation time. This is probably due to the difference in properties between tryptophan and glycine as discussed in the HOPE results, especially glycine being more flexible than tryptophan. This is also in line with the CASTp 3.0 results where this mutation massively altered the largest protein binding pocket and was predicted to impact ligand binding. However, towards the end of the simulation time, the 5-turn helix conformation was replaced by turns. It is still very much possible that ultimately the conformation of that region would be a 5-turn helix in the W33G system, as that was the conformation conferred on the protein for the majority of the simulation time. The region in question covers three ligand binding sites at residues 26, 29, and 33. Hence, such a change could drastically impact ligand binding. The W33G system also brought back the α-helix conformation observed in the apo form of the protein between residues 222 and 237, which was absent in the WT-SAH system. As observed in prior analyses, W33G tends to enhance the plasticity of the protein. This was also observed in the variation of the secondary structure across time, wherein W33G had far lesser variation in the secondary structure conformations over time when compared to the WT-SAH system.

W78R on the other hand did introduce some more secondary structure variation in the initial residues and close to the mutation site, but reduced variation in other places. Furthermore, the α-helix between residues 222 and 237 as seen in the W33G system, and the apo form of the protein was also seen in the first 50 ns in the W78R system, post which it was largely replaced by turns. Similar to W78R, the V89E system seemed to reduce the structural variation when compared to the WT-SAH system, although not to the degree observed in the W33G system, probably due to the enhanced plasticity caused by the V89E mutation. Notably, the α-helix conformation near residue 137 as observed in the WT-SAH system for a majority of the simulation time, was absent from the V89E system. This could be problematic for ligand binding as the region near residue 133 contains three ligand binding sites at residues 133, 134 and 135. The α-helix between residues 222 and 237 did appear in the first 5 ns in the V89E system as well, however it was replaced by bends after that. W150G appeared to have a slightly increased variation in the secondary structure when compared to the WT-SAH system which is in line with prior MDS analyses where W150G was predicted to impart instability to the system. It also introduced a 5-turn helix conformation between residues 47 and 57 as opposed to an α-helix conformation in the WT-SAH system. The α-helix between residues 222 and 237 also appeared in the W150G system, albeit only for the first 5 ns similar to V89E post which it only appeared on a few occasions. Curiously, L182P also created an α-helix conformation between residues 222 and 237, which lasted for over 60 ns in the 100 ns of simulation time. Akin to other mutations, the L182P system imparted enhanced plasticity to the protein and as a result, showed a reduced variation in secondary structure conformations across the simulation time to a degree similar to that observed in the W33G system. This lines up with the PCA results where L182P showed a restricted collective motion to a degree similar to W33G.

This common trend of introducing the α-helix conformation between residues 222 and 237 in mutant systems could be due to the fact that its presence might be partially responsible for enhanced plasticity. Upon closer inspection, it was found that while this residue region was in fact not in the α-helix conformation in the 100 ns simulation time of the WT-SAH system, it was in the α-helix conformation in the final ligand-bound TPMT structure i.e., in the structure with PDB ID 2H11. Furthermore, this particular stretch of residues harbored 28 hydrogen bonds in the final structure. Figure S7 (generated using UCSF Chimera^98^) shows the hydrogen bonds present in the final ligand-bound TPMT structure (2H11) between the stretch of residues from residue 221 to residue 237. This suggests that the formation of the α-helix conformation between residues 222 and 237 occurs post 100 ns and is important for enhancing the stability of the protein, as it is present in the final conformation. As the mutations increase plasticity, it is logical to surmise that they might introduce this conformation very early in order to contribute to the plasticity of the protein.

### Binding energy analysis

To further analyze the impact of the missense mutations on the protein with respect to ligand binding, the MM-PBSA method was used over the last 70 ns of simulation time with an interval of 100 ps. Various components of binding energy including van der Waals energy, electrostatic energy, polar solvation energy and SASA energy, as well as the binding energy itself, which is the cumulative sum of the aforementioned components, were calculated for all systems except the apo form of TPMT. The average values of these parameters are stated in Table 5. It was observed that apart from L182P, all mutations drastically increased the binding free energy when compared to ligand-bound TPMT with W33G having the most significant impact on binding free energy. These results are in line with prior MDS analyses where most mutations enhanced the plasticity of the protein which has likely hampered ligand binding as evident from the binding free energy values. Furthermore, these results are also in line with the secondary structure analysis and PCA results analysis where it was observed that W33G seemed to impart the most plasticity to the protein–ligand system.

**Table 5:** Binding energy and its components including van der Waals, electrostatic, polar solvation and SASA energy for all six ligand-bound systems, calculated over the last 70 ns of simulation time.

System	Van der Waals energy (kJ/mol)	Electrostatic energy (kJ/mol)	Polar solvation energy (kJ/mol)	SASA energy (kJ/mol)	Binding energy (kJ/mol)
WT-SAH	− 215.76 ± 11.86	− 47.82 ± 9.34	146.29 ± 12.92	− 21.48 ± 0.92	− 138.77 ± 14.68
W33G	− 0.004 ± 0.00	0.00 ± 0.00	16.21 ± 50.22	− 0.40 ± 3.47	15.81 ± 50.25
W78R	− 0.005 ± 0.00	0.00 ± 0.00	5.13 ± 61.70	− 0.47 ± 3.52	4.65 ± 61.61
V89E	− 0.005 ± 0.00	0.00 ± 0.00	− 17.84 ± 47.13	0.39 ± 3.64	− 17.45 ± 47.15
W150G	− 0.005 ± 0.00	0.00 ± 0.00	− 28.27 ± 41.29	− 0.52 ± 3.85	− 28.8 ± 41.57
L182P	− 210.10 ± 11.34	− 60.55 ± 10.00	189.25 ± 14.48	− 20.84 ± 0.87	− 102.24 ± 13.42

**Figure 5:**
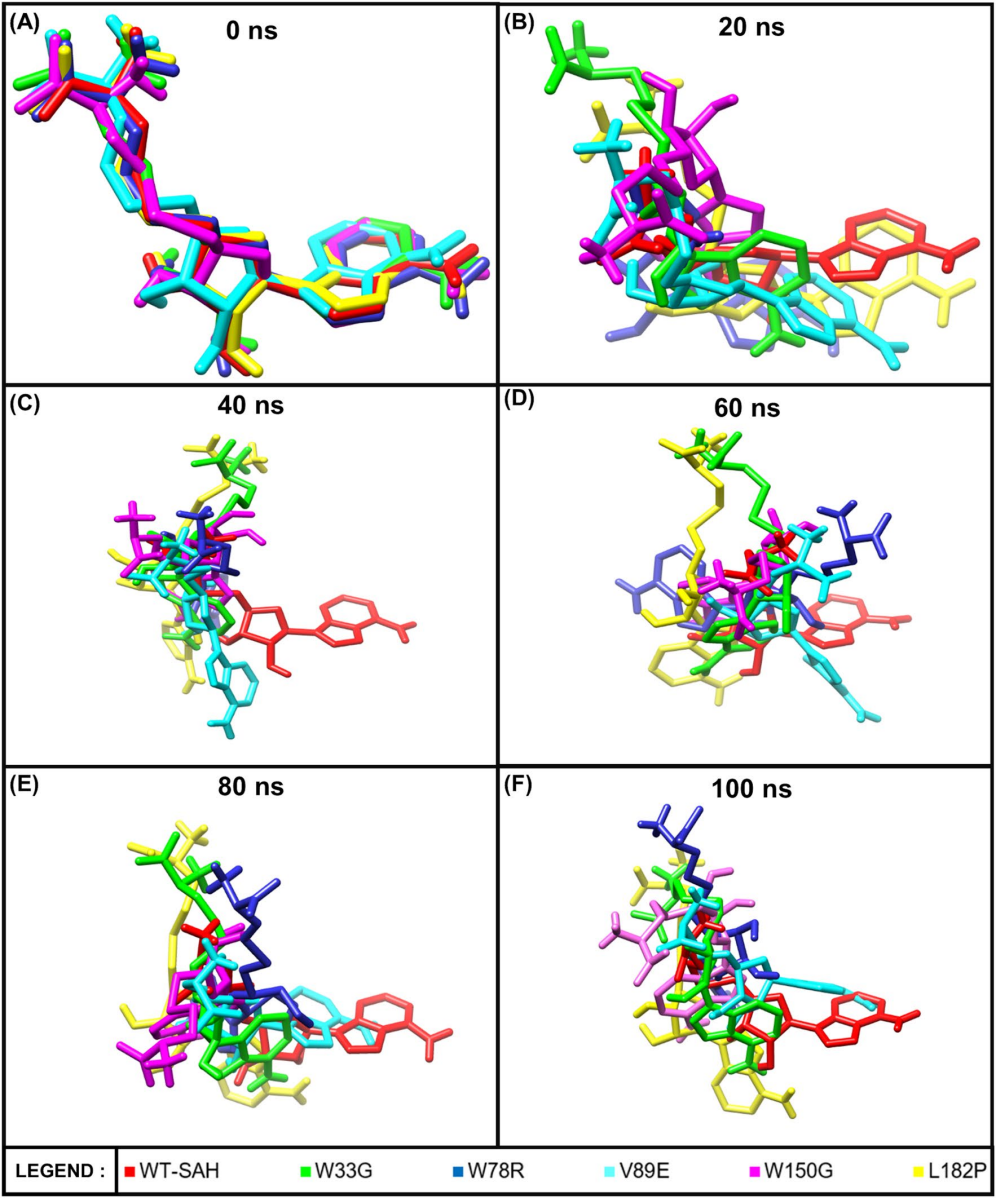
Ligand conformations for all six ligand-bound systems across 0, 20, 40, 60, 80 and 100 ns of simulation time.

A graph showcasing the residual contribution to binding energy at catalytic residues (residue number 26, 29, 33, 40, 69, 70, 90, 91, 133, 134, 135, 152 and 153) involved in ligand binding is presented in Figure S8. As is evident from the figure, most catalytic residues were unable to contribute to binding energy in all mutations except L182P. Interestingly, L182P shows a nearly threefold increase in binding energy at residue 152 when compared to wild-type ligand-bound TPMT. As shown in Figure S9, in the wild-type ligand-bound TPMT (WT-SAH) system the distance between residue 152 and the ligand is over 7 Å while in the L182P mutant system, the distance between residue 152 and the ligand is just over 3 Å. Furthermore, there are a lot of hydrogen bonds nearby, making the conformations very rigid as opposed to a single one observed at the ligand near residue 152 in the case of the WT-SAH system. This increased proximity and rigidity may cause repulsion and result in a threefold increase in binding energy at residue 152, as observed in the case of L182P. Figure 5 shows the change in ligand conformations over the simulation time for all six ligand-bound systems. It can be clearly seen that the ligand conformations are very similar at the start of the simulation (0 ns) and then from 20 ns onwards, they show variation with respect to the WT-SAH system. Animation 10 provides a 360° view of the change in ligand conformations over the simulation time for all six ligand-bound systems.

### Relative deleterious nature of the screened deleterious TPMT missense SNPs

**Figure 6:**
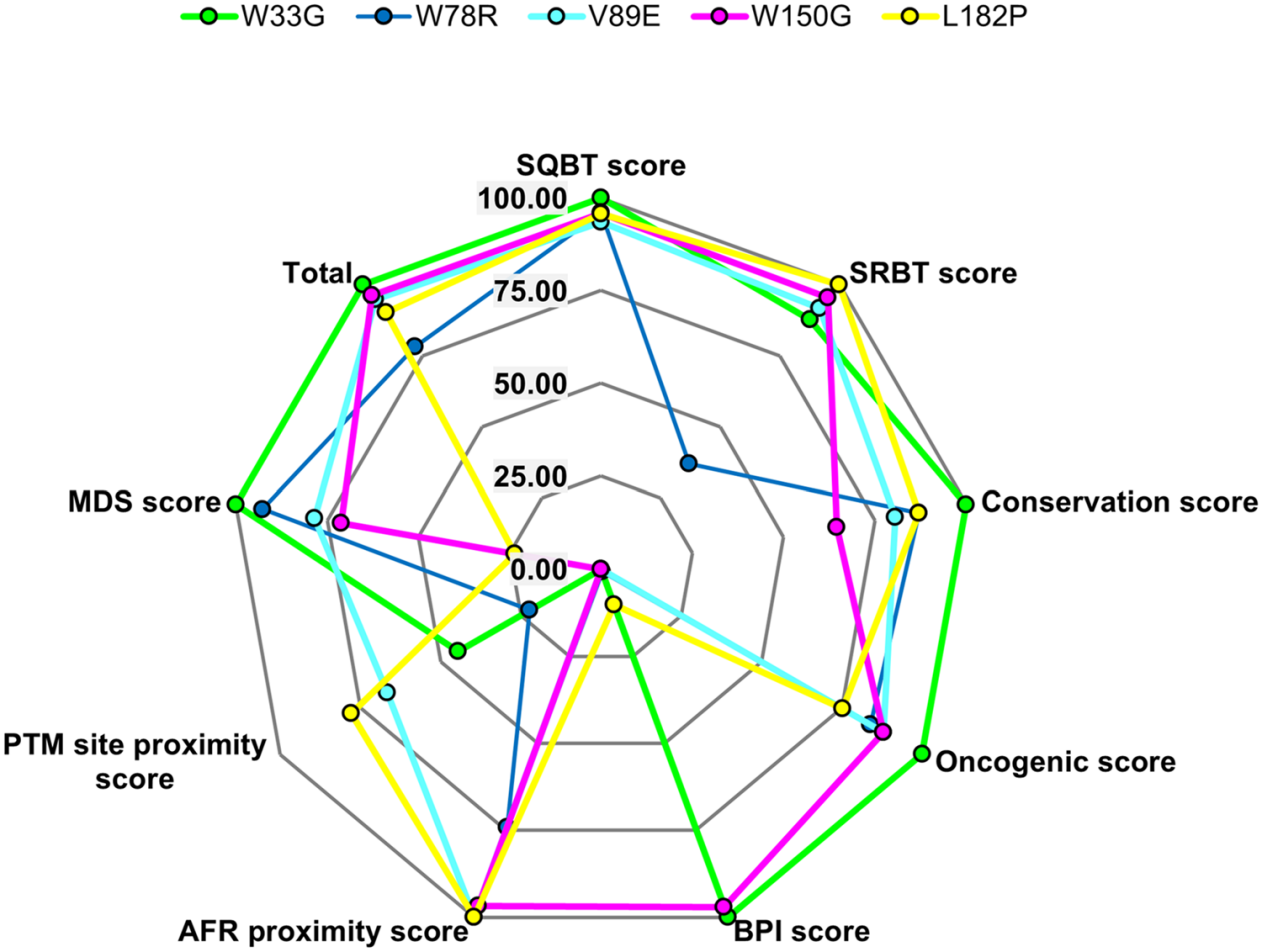
The relative deleterious nature of the five highly deleterious TPMT missense SNPs. A score of 100 for a mutation in a particular category indicates that the given mutation is the most deleterious with respect to that category (SQBT = Sequence-based tools, SRBT = Structure-based tools, BPI score = Binding pocket impact score, AFR = Amyloid forming region, PTM = Post-translational modification, MDS = Molecular dynamics simulations).

To summarise the present study and visually draw meaningful conclusions, the relative deleterious natures of the five highly deleterious missense SNPs were determined, as shown in Fig. 6. The relative deleterious natures of the five nsSNPs were calculated through their respective absolute cumulative scores of sequence-based and structure-based tools, cumulative conservation scores at the mutated residues by ConSurf, CDD and all MSA tools, cumulative coding scores provided by both CScape and CScape-somatic (Oncogenic scores), changes in protein binding pockets with respect to volume (cumulative for W33G and L182P, due to the presence of multiple binding pocket changes), the distances to nearest amyloid-forming regions, the distances to nearest PTM sites, and the respective binding energies of the mutant systems calculated using the MM-PBSA method (MDS score) during MDS analysis.

Consider the oncogenic score where the cumulative score of CScape and CScape-somatic were considered. W33G was observed to have the highest cumulative oncogenic score among all mutations. Hence, to calculate the relative oncogenic score of say W78R, the cumulative oncogenic score of W78R (1.806) was divided by the cumulative oncogenic score of W33G (1.516) and multiplied by 100 which resulted in a relative oncogenic score for W78R as 83.942%.

This procedure was followed for all of the aforementioned categories for all mutations in order to unify the scale for all categories for all mutations for better interpretation and visualization. Due to the nature of the procedure, a score of 100 for a mutation in a given category implies that it has the most deleterious effect on TPMT with respect to that category when compared to the other four mutations. For example, W33G has an oncogenic score of 100, which implies that it is the most deleterious missense SNP of TPMT among the five screened mutations with respect to oncogenicity. It is worth noting that for the categories that account for the distance of mutations from their nearest PTM site and nearest amyloid-forming region, a very minor change was incorporated into the methodology. In the case of those two categories, after following the same aforementioned methodology to get the relative score, it was subtracted from 100 to get the final relative score. This was done because contrary to the other categories, a lower value indicates a closer distance to the nearest PTM site or amyloid-forming region which implies that the given mutation has a more deleterious effect. Finally, a category named “Total” was computed where the relative scores of all categories were added and then using the same procedure used in the other categories, the “Total” category itself was calculated relative to the highest value in the “Total” category (W33G). Thus, from Fig. 6 it can be seen that W33G is the most deleterious missense mutation of TPMT amongst the five deleterious SNPs followed by W150G, V89E, L182P and W78R in the order of decreasing deleterious nature.

## Conclusion

The missense SNPs of the TPMT gene have been analyzed in the present study in order to identify novel deleterious SNPs through in silico means, including the validation of the in silico screening pipeline. TPMT was chosen due to the known association of its polymorphisms with the disease progression and treatments of several dangerous diseases including leukemia and IBD (Crohn's disease and ulcerative colitis) (IBD). Since it is a known fact that in silico protocols cannot completely replace in vitro and in vivo experimental protocols which are usually more conclusive in nature, the present study has attempted to validate the screening pipeline by means of re-identifying known deleterious alleles of TPMT through the same. This was followed by the utilisation of a plethora of tools and analyses to gain a more comprehensive understanding of how the SNPs identified in the screening phase might be deleterious in nature. Among all missense SNPs of TPMT reported in the dbSNP database so far, five novel highly deleterious SNPs namely W33G (rs72552741), W78R (rs753277177), V89E (rs1784191846), W150G (rs1447033392) and L182P (rs1386533390) were identified in the present study. These SNPs have a high probability of being associated with the aforementioned diseases that are known to be associated with TPMT polymorphisms. The most deleterious SNP among the five was found to be W33G based on several different analyses. The authors believe that by giving a higher priority to these particular SNPs rather than considering the massive pool of all TPMT SNPs, future in vivo or in vitro research endeavors targeted at TPMT polymorphisms and/or their consequences in relevant disease progressions or treatments will greatly benefit from the present study.

## Supplementary Information

Supplementary Information 1.

Supplementary Information 2.

Supplementary Information 3.

Supplementary Video 1.

Supplementary Video 2.

Supplementary Video 3.

Supplementary Video 4.

Supplementary Video 5.

Supplementary Video 6.

Supplementary Video 7.

Supplementary Video 8.

## Data Availability

The SNP data utilized in this study are openly available in the dbSNP database at https://www.ncbi.nlm.nih.gov/snp/?term=TPMT. The STRING database and CDD (Conserved Domain Database), used in the present study are available at https://string-db.org/ and https://www.ncbi.nlm.nih.gov/cdd/ respectively. The protein sequence and structure used for TPMT in the present study, obtained from the UniProt database and PDB (Protein Data Bank) respectively are available at https://www.uniprot.org/uniprotkb/P51580/entry#sequences and https://www.rcsb.org/structure/2H11 respectively. All data generated in the present study is present in the article and supplementary information.
