# Supplementary material for: A bioinformatics approach to the identification of novel deleterious mutations of human TPMT through validated screening and molecular dynamics
Source: Sci Rep. 2022 Nov 7;12:18872. doi: 10.1038/s41598-022-23488-z (PMC9640560; doi:10.1038/s41598-022-23488-z)
Supplement: Supplementary file 1 — Supplementary Information 1. [file 41598_2022_23488_MOESM1_ESM.docx]

***Supplementary information for***

**Comprehensive *in silico* analysis of Human TPMT missense mutations and validation of the *in silico* screening pipeline through known deleterious TPMT alleles**

Sidharth Saxena^1^, T P Krishna Murthy^1*^, Chandrashekar CR^1^, Lavan S Patil^1^, Abhinav Aditya^1^, Rohit Shukla^2^, Arvind Kumar Yadav^2^, Tiratha Raj Singh^2^, Mahesh Samantaray^3^, R Amutha^3^

^1^Department of Biotechnology, Ramaiah Institute of Technology, Bengaluru-560054, Karnataka, India

^2^Department of Biotechnology and Bioinformatics, Jaypee University of Information Technology (JUIT), Solan-173234, Himachal Pradesh, India

^3^Department of Bioinformatics, Pondicherry University, Pondicherry - 605 014, India

***Corresponding author**

***Dr. Krishna Murthy T P****, PhD*

Assistant Professor

Department of Biotechnology

M S Ramaiah Institute of Technology

Bengaluru-560054, Karnataka, INDIA

**Email:** [tpk@live.in](mailto:tpk@live.in)

**ORCID ID:** 0000-0002-9533-7567

**FIGURES**


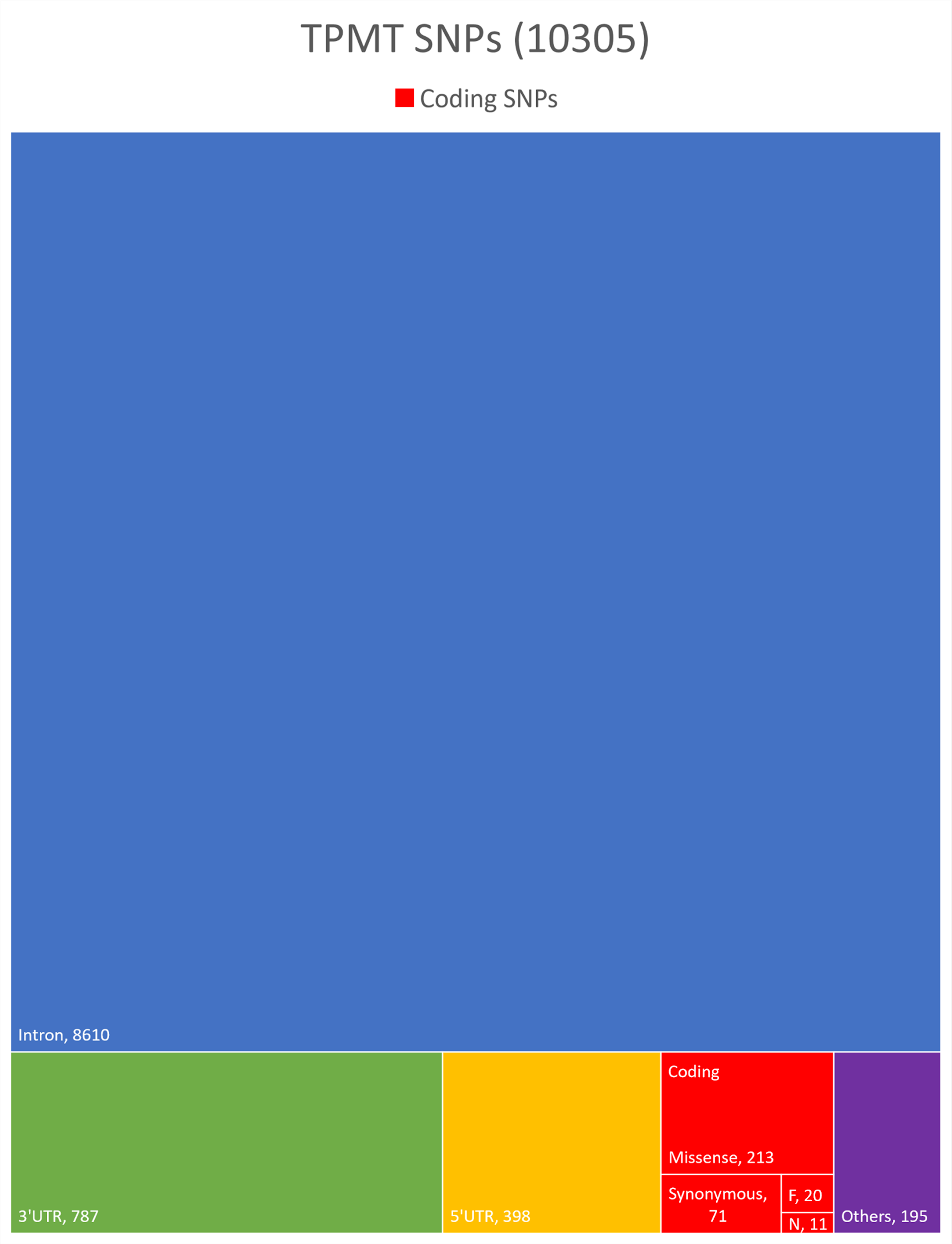


**Figure S1 :** Distribution of TPMT SNPs (F=Frameshift mutations, N=Nonsense mutations).

**
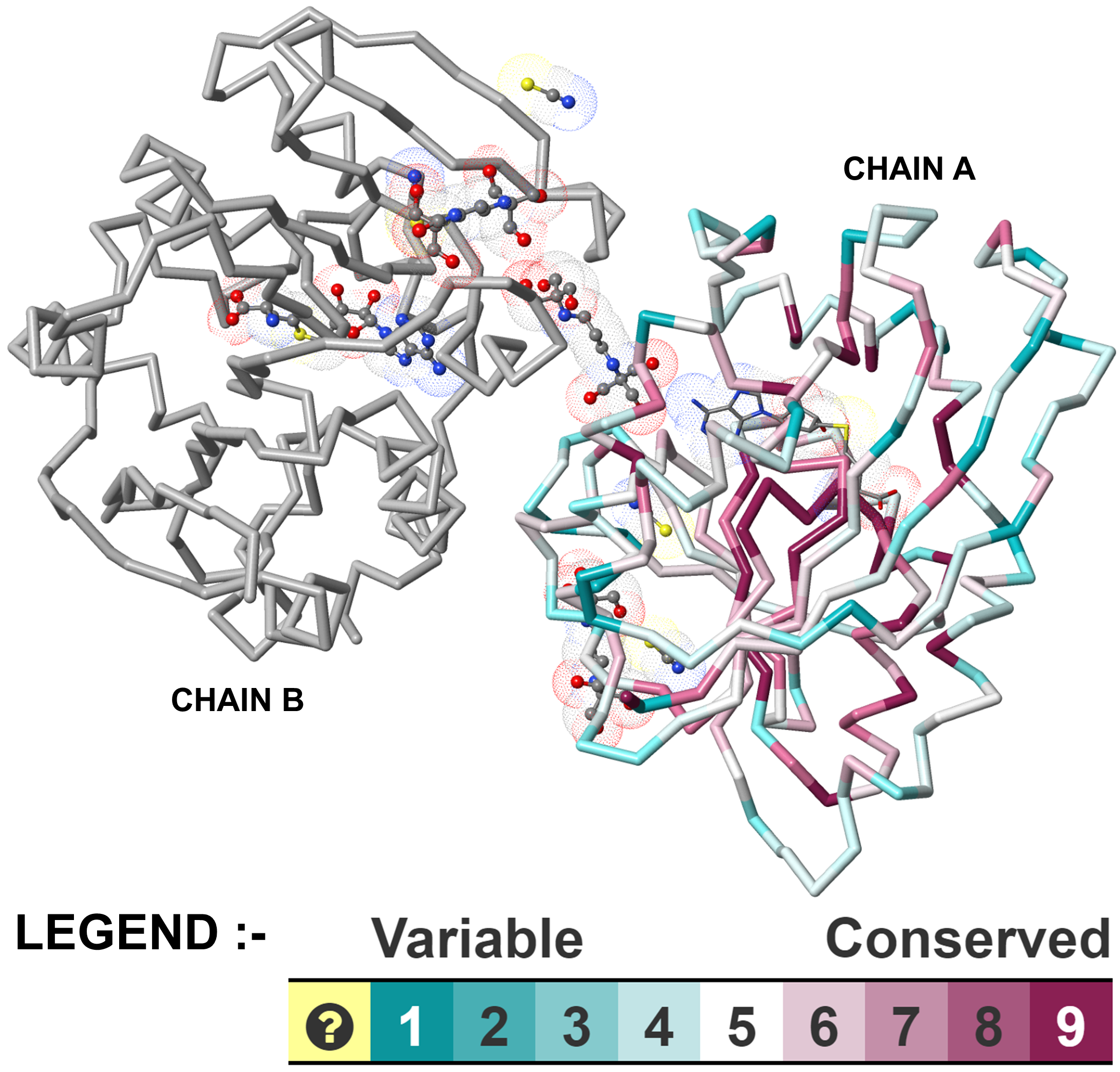
**

**Figure S2 :** Conservation of chain A of TPMT (2H11) as predicted by ConSurf server.

**
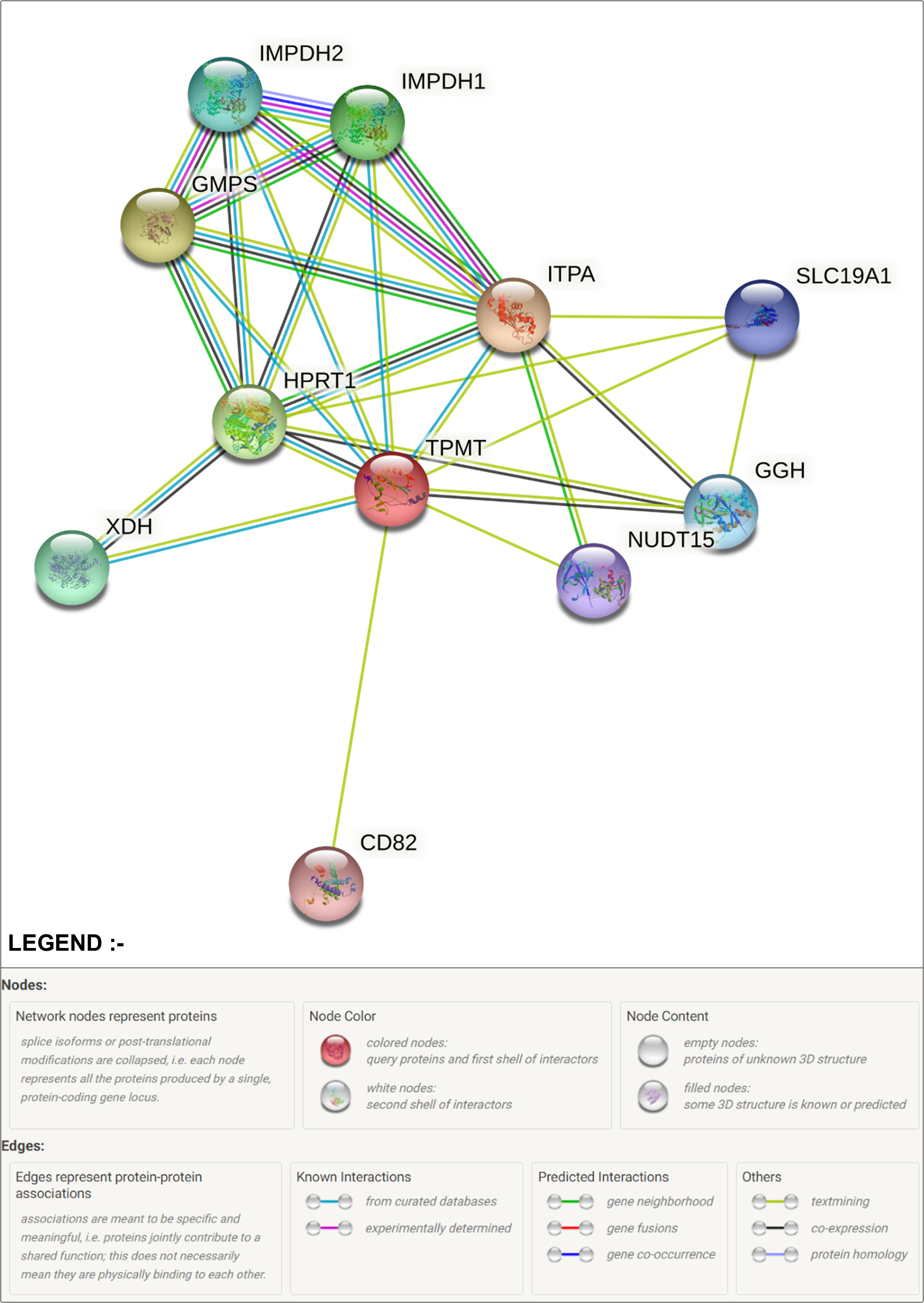
**

**Figure S3 :** Interaction network of TPMT, obtained from the STRING database.


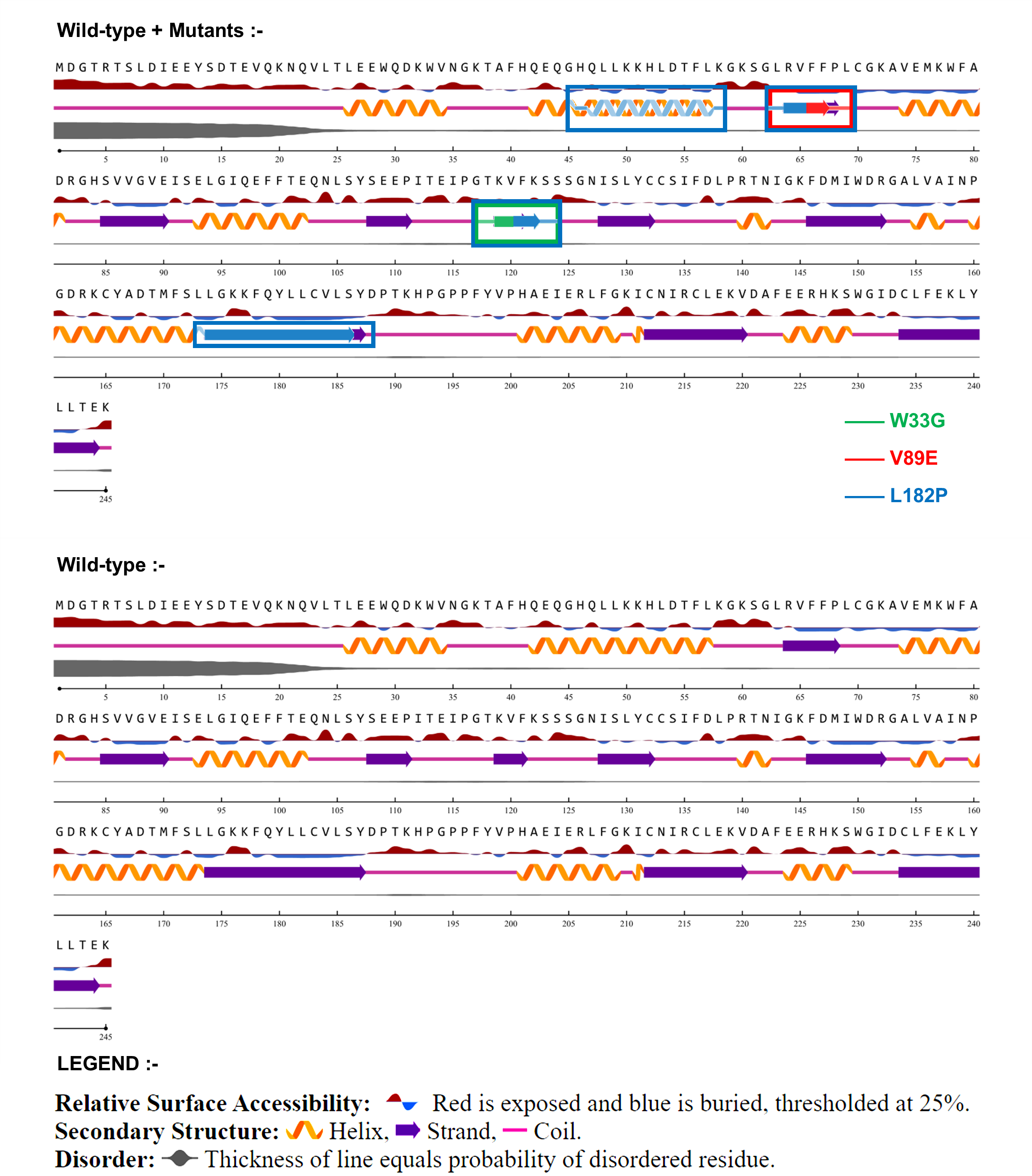


**Figure S4 :** Secondary structure alterations caused by the mutations, as predicted by NetSurfP-2.0.

**
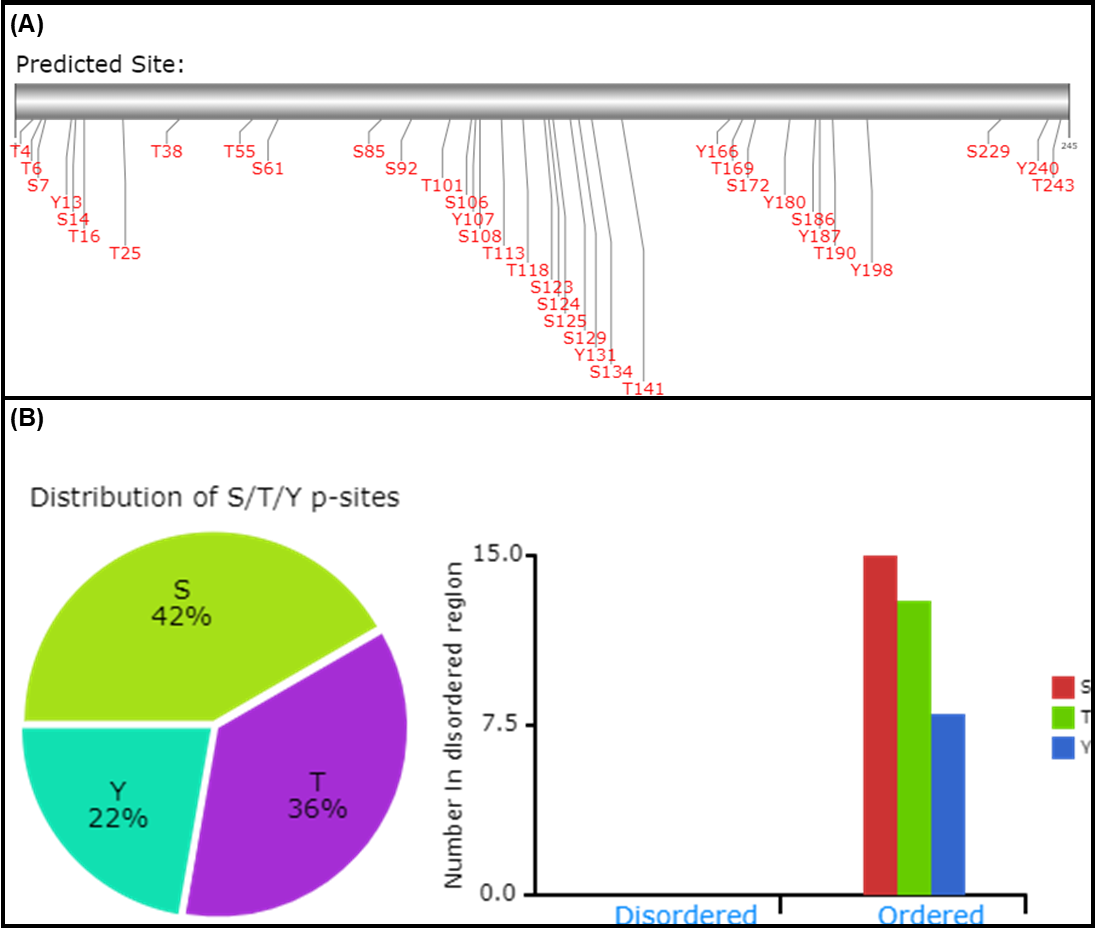
**

**Figure S5 :** Prediction of PTM sites and their distribution, obtained from GPS 5.0.


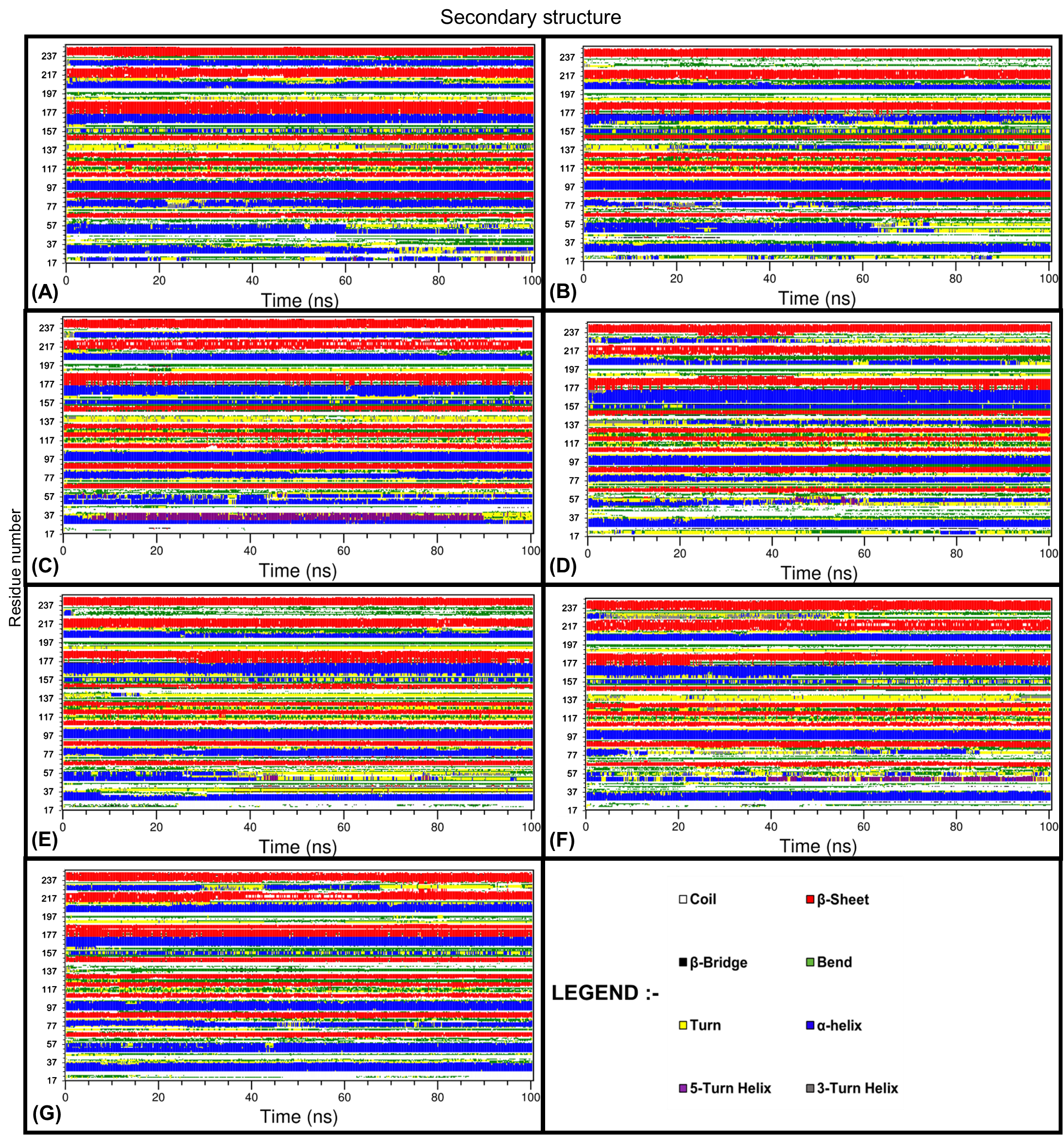


**Figure S6 :** Secondary structure analysis results (the first 16 residues were not modelled in the reference structure (2H11), hence the residue numbers start from residue 17).


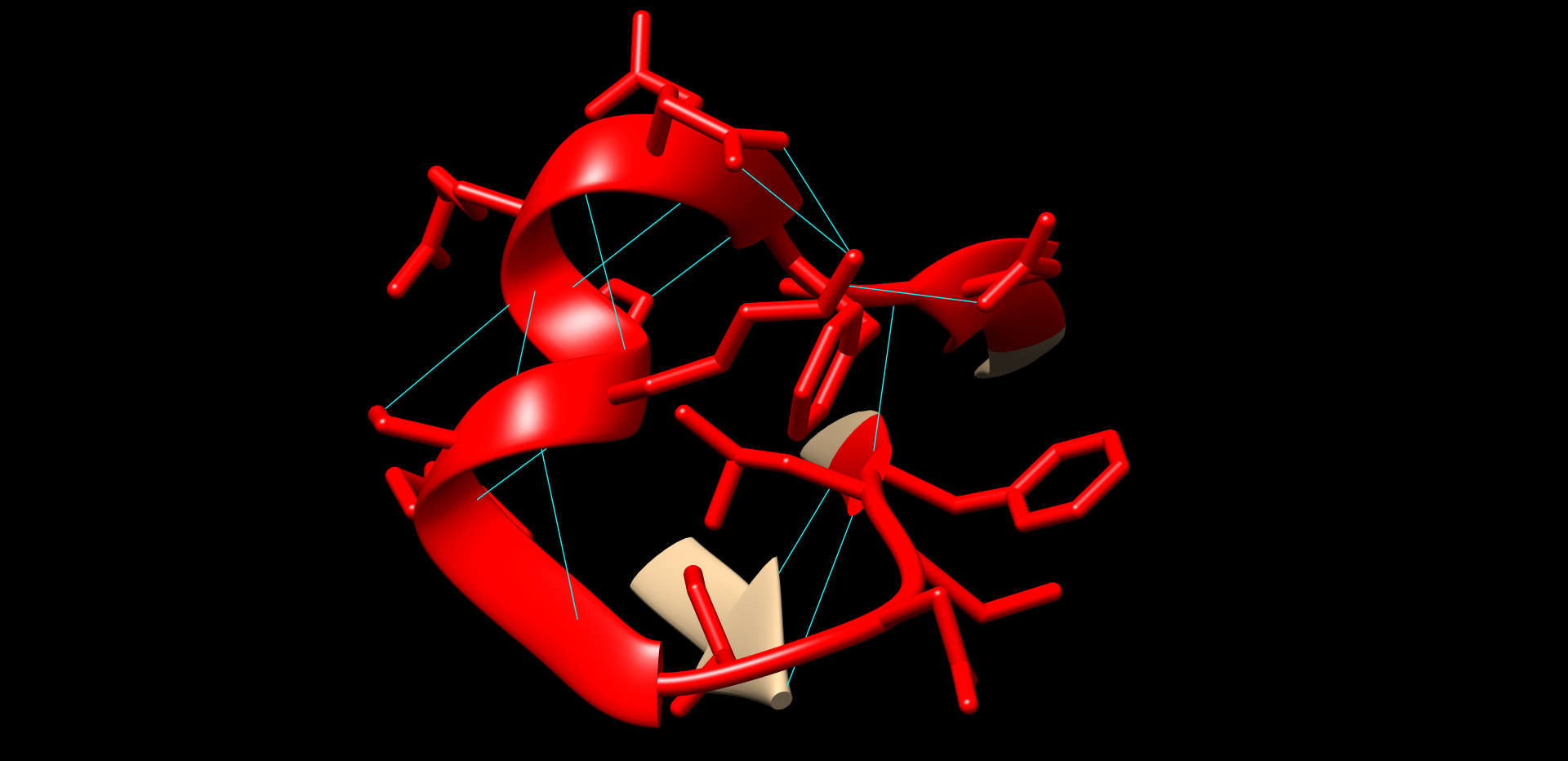


**Figure S7 :** Hydrogen bonds (represented in blue) present in the final ligand-bound TPMT structure (2H11) between the stretch of residues from residue 221 to residue 237 (represented in red).

**
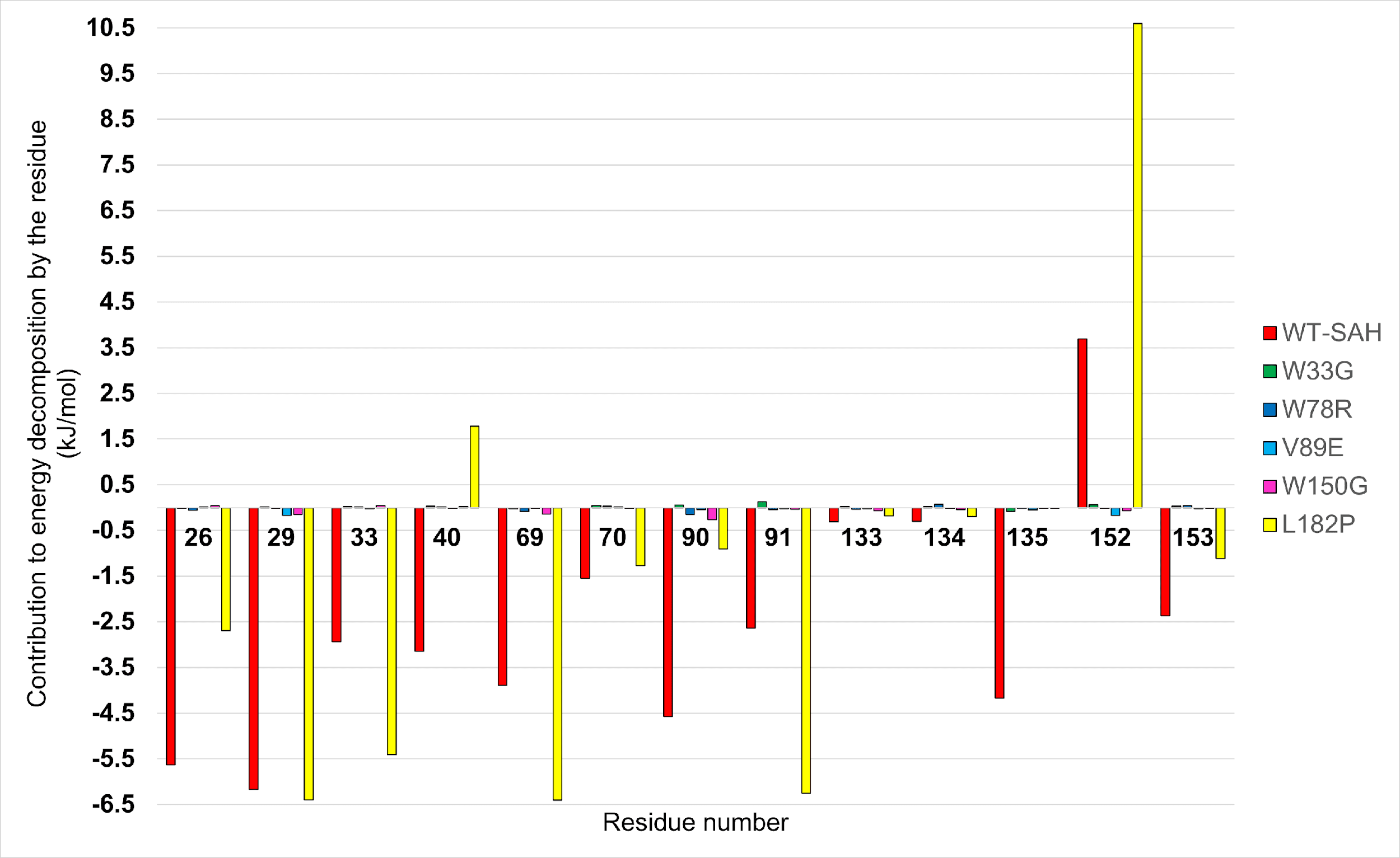
**

**Figure S8 :** Prediction of PTM sites and their distribution, obtained from GPS 5.0.


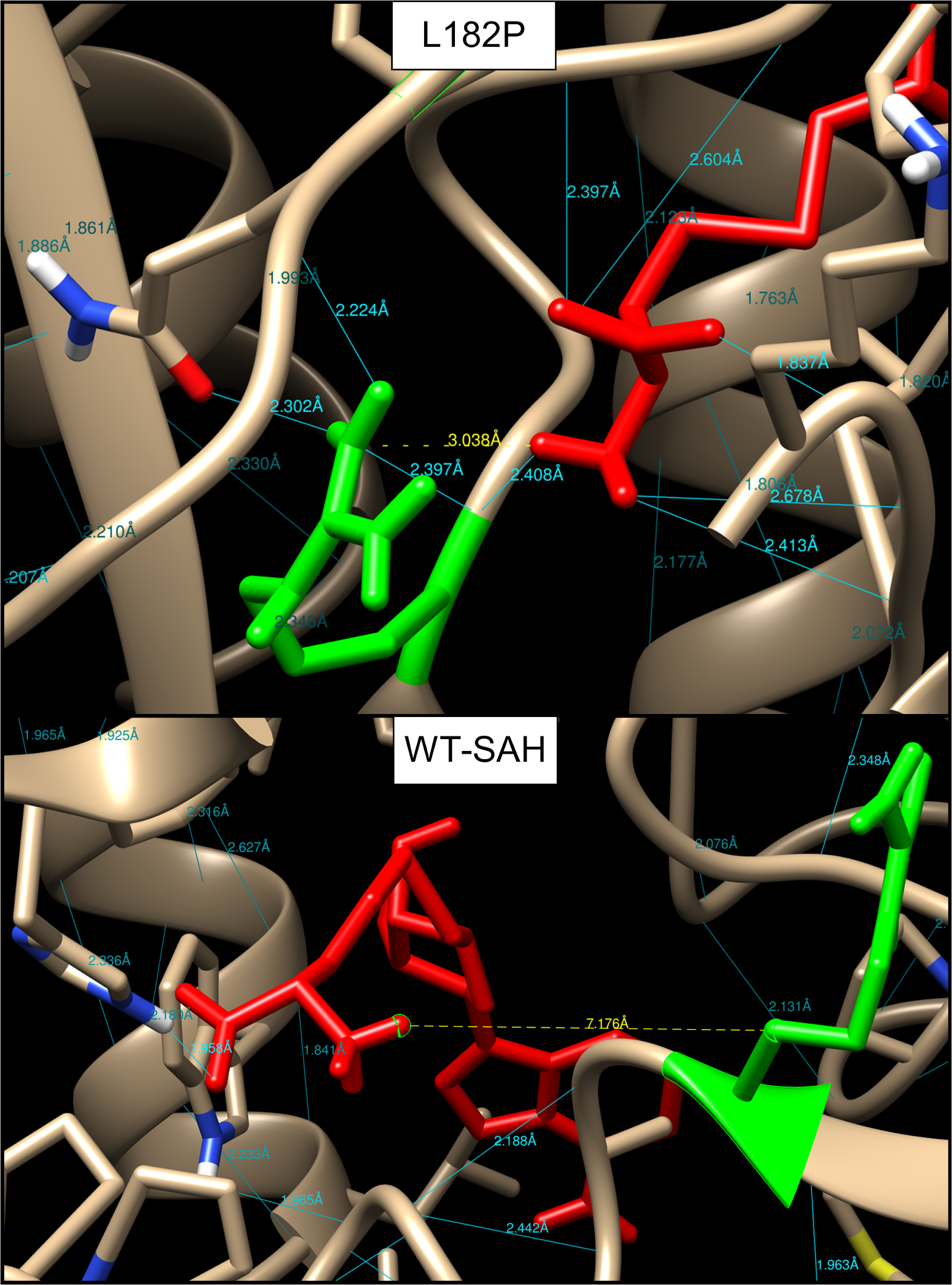


**Figure S9 :** Difference in the ligand and residue 152 environment between the L182P mutant system and WT-SAH system (Ligand is coloured red, residue 152 is coloured green, the distance between the ligand and residue 152 is coloured yellow and the hydrogen bonds are represented in blue colour).

**TABLES**

**Table S1:** Predictions for all missense nsSNPs obtained from dbSNP for TPMT by sequence-based tools with their respective scores.

Data has been provided in the excel file.

**Table S2:** Predictions by structure-based tools with their respective scores for the 52 missense nsSNPs obtained after screening through sequence-based tools.

Data has been provided in the excel file.

**Table S3:** Predictions made by sequence-based tools, structure-based tools, MSA tools and ConSurf for pre-identified SNPs.

Data has been provided in the excel file.

**Table S4:** Conservation analysis results predicted by ConSurf for all residues of TPMT (2H11).

Data has been provided in the excel file.

**Table S5:** Conservation analysis results predicted by MSA tools, ConSurf and Conserved Domain Database (CDD) for the six missense nsSNPs obtained after screening through sequence-based and structure-based tools (The range of the possible scores for each tool are mentioned next to the tool name).

| **Variant ID** | **SNP** |  | **MSA (1-10)** | | | | |  | **ConSurf (1-9)** | |  | **CDD (1-13)** |
| --- | --- | --- | --- | --- | --- | --- | --- | --- | --- | --- | --- | --- |
|  |  |  | **Clustal Omega** | **Kalign** | **T-Coffee** | **MAFFT** | **MUSCLE** |  | **Score** | **Colour score** |  | **Score** |
| rs72552741 | W33G |  | 10 | 10 | 10 | 10 | 10 |  | -1.561 | 9 |  | 12 |
| rs753277177 | W78R |  | 10 | 10 | 10 | 10 | 10 |  | -1.284 | 9 |  | 8 |
| rs1784191846 | V89E |  | 10 | 10 | 10 | 10 | 10 |  | -1.072 | 8 |  | 7 |
| rs1447033392 | W150G |  | 10 | 10 | 10 | 10 | 10 |  | -1.019 | 8 |  | 2 |
| rs1386533390 | L182P |  | 9 | 9 | 9 | 9 | 9 |  | -0.184 | 6 |  | 12 |

**Table S6:** All functional partners of TPMT along with their respective scores as predicted by the STRING database.

| Functional Partner | Score | Information |
| --- | --- | --- |
| ITPA | 0.979 | Inosine triphosphate pyrophosphatase. It is a pyrophosphatase that hydrolyzes the non-canonical purine nucleotides inosine triphosphate (ITP), deoxyinosine triphosphate (dITP) as well as 2'-deoxy-N-6-hydroxylaminopurine triposphate (dHAPTP) and xanthosine 5'-triphosphate (XTP) to their respective monophosphate derivatives. The enzyme does not distinguish between the deoxy- and ribose forms and probably excludes non-canonical purines from RNA and DNA precursor pools, thus preventing their incorporation into RNA and DNA and avoiding chromosomal lesions. |
|  |  |  |
| GMPS | 0.948 | GMP synthase [glutamine-hydrolyzing]. It is involved in the de novo synthesis of guanine nucleotides which are not only essential for DNA and RNA synthesis, but also provide GTP, which is involved in a number of cellular processes important for cell division. |
|  |  |  |
| HPRT1 | 0.947 | Hypoxanthine-guanine phosphoribosyltransferase. It converts guanine to guanosine monophosphate, and hypoxanthine to inosine monophosphate. It also transfers the 5- phosphoribosyl group from 5-phosphoribosylpyrophosphate onto the purine. It plays a central role in the generation of purine nucleotides through the purine salvage pathway. |
|  |  |  |
| IMPDH1 | 0.944 | Inosine-5'-monophosphate dehydrogenase 1. It catalyzes the conversion of inosine 5'-phosphate (IMP) to xanthosine 5'-phosphate (XMP), the first committed and rate-limiting step in the de novo synthesis of guanine nucleotides, and therefore plays an important role in the regulation of cell growth. It could also have a single-stranded nucleic acid-binding activity and could play a role in RNA and/or DNA metabolism. It may also have a role in the development of malignancy and the growth progression of some tumors. |
|  |  |  |
| XDH | 0.937 | Xanthine dehydrogenase/oxidase. It is a key enzyme in purine degradation, which catalyzes the oxidation of hypoxanthine to xanthine and xanthine to uric acid. It contributes to the generation of reactive oxygen species and also has low oxidase activity towards aldehydes (in vitro). |
|  |  |  |
| IMPDH2 | 0.927 | Inosine-5'-monophosphate dehydrogenase 2. Similar to IMPDH1, it catalyzes the conversion of inosine 5'-phosphate (IMP) to xanthosine 5'-phosphate (XMP). IMPDH1 and IMPDH2 are isozymes, both of which contain the same number of residues and are 84% similar with respect to their peptide sequences. They also share similar kinetic properties. |
|  |  |  |
| GGH | 0.910 | Gamma-glutamyl hydrolase. It hydrolyzes the polyglutamate sidechains of pteroylpolyglutamates and progressively removes gamma-glutamyl residues from pteroylpoly-gamma-glutamate to yield pteroyl-alpha- glutamate (folic acid) and free glutamate. It may play an important role in the bioavailability of dietary pteroylpolyglutamates and in the metabolism of pteroylpolyglutamates and antifolates. |
|  |  |  |
| SLC19A1 | 0.908 | Folate transporter 1. It is a transporter for the intake of folate. Uptake of folate in human placental choriocarcinoma cells occurs by a novel mechanism called potocytosis which functionally couples three components, namely the folate receptor, the folate transporter, and a V-type H(+)-pump. |
|  |  |  |
| NUDT15 | 0.870 | Nucleotide triphosphate diphosphatase. It may catalyze the hydrolysis of nucleoside triphosphates including dGTP, dTTP, dCTP, their oxidized forms like 8-oxo-dGTP and the prodrug thiopurine derivatives 6-thio-dGTP and 6-thio-GTP. It could also catalyze the hydrolysis of some nucleoside diphosphate derivatives. It hydrolyzes oxidized nucleosides triphosphates like 8-oxo-dGTP in vitro, but the specificity and efficiency towards these substrates are low. Therefore, the potential in vivo sanitizing role of this enzyme, that would consist in removing oxidatively damaged forms of nucleosides to prevent their incorporation into DNA, is unclear. Through the hydrolysis of thioguanosine triphosphates, it may participate in the catabolism of thiopurine drugs. It may also have a role in DNA synthesis and cell cycle progression by stabilizing PCNA. |
|  |  |  |
| CD82 | 0.804 | CD82 antigen. It associates with CD4 or CD8 and delivers costimulatory signals for the TCR/CD3 pathway. |

**Table S7:** Biological processes which the TPMT interaction network is involved in, obtained using the STRING database.

| GO Accession | Biological Processes | Gene Count | Strength | False Discovery Rate | Matching Proteins in the Network |
| --- | --- | --- | --- | --- | --- |
| GO:0006177 | GMP biosynthetic process | 4 out of 4 | 3.25 | 3.30E-08 | HPRT1,IMPDH2,IMPDH1,GMPS |
| GO:0009146 | purine nucleoside triphosphate catabolic process | 2 out of 6 | 2.77 | 0.0022 | NUDT15,ITPA |
| GO:0009204 | deoxyribonucleoside triphosphate catabolic process | 2 out of 8 | 2.65 | 0.0035 | NUDT15,ITPA |
| GO:0006183 | GTP biosynthetic process | 2 out of 11 | 2.51 | 0.0057 | IMPDH2,IMPDH1 |
| GO:0009113 | purine nucleobase biosynthetic process | 2 out of 12 | 2.47 | 0.0065 | HPRT1,GMPS |
| GO:0006144 | purine nucleobase metabolic process | 3 out of 22 | 2.38 | 0.00013 | HPRT1,XDH,GMPS |
| GO:0006195 | purine nucleotide catabolic process | 4 out of 43 | 2.22 | 7.92E-06 | NUDT15,HPRT1,XDH,ITPA |
| GO:0006760 | folic acid-containing compound metabolic process | 2 out of 27 | 2.12 | 0.0247 | GGH,SLC19A1 |
| GO:0034404 | nucleobase-containing small molecule biosynthetic process | 7 out of 105 | 2.07 | 9.40E-10 | NUDT15,HPRT1,IMPDH2,IMPDH1,XDH,ITPA,GMPS |
| GO:0009154 | purine ribonucleotide catabolic process | 2 out of 30 | 2.07 | 0.0294 | HPRT1,ITPA |
| GO:0009144 | purine nucleoside triphosphate metabolic process | 4 out of 69 | 2.01 | 3.15E-05 | NUDT15,IMPDH2,IMPDH1,ITPA |
| GO:0009205 | purine ribonucleoside triphosphate metabolic process | 3 out of 61 | 1.94 | 0.0016 | IMPDH2,IMPDH1,ITPA |
| GO:0046651 | lymphocyte proliferation | 3 out of 92 | 1.81 | 0.0035 | HPRT1,IMPDH2,IMPDH1 |
| GO:0006163 | purine nucleotide metabolic process | 7 out of 329 | 1.58 | 7.48E-07 | NUDT15,HPRT1,IMPDH2,IMPDH1,XDH,ITPA,GMPS |
| GO:0009150 | purine ribonucleotide metabolic process | 5 out of 303 | 1.47 | 0.00016 | HPRT1,IMPDH2,IMPDH1,ITPA,GMPS |
| GO:1901136 | carbohydrate derivative catabolic process | 3 out of 183 | 1.46 | 0.0278 | NUDT15,HPRT1,ITPA |
| GO:1901135 | carbohydrate derivative metabolic process | 6 out of 987 | 1.03 | 0.0018 | NUDT15,HPRT1,IMPDH2,IMPDH1,ITPA,GMPS |
| GO:0044281 | small molecule metabolic process | 9 out of 1684 | 0.98 | 9.06E-06 | NUDT15,GGH,HPRT1,SLC19A1,IMPDH2,IMPDH1,XDH,ITPA,GMPS |
| GO:0046483 | heterocycle metabolic process | 10 out of 2840 | 0.8 | 2.43E-05 | NUDT15,GGH,HPRT1,SLC19A1,TPMT,IMPDH2,IMPDH1,XDH,ITPA,GMPS |
| GO:0006725 | cellular aromatic compound metabolic process | 10 out of 2882 | 0.79 | 2.67E-05 | NUDT15,GGH,HPRT1,SLC19A1,TPMT,IMPDH2,IMPDH1,XDH,ITPA,GMPS |
| GO:1901360 | organic cyclic compound metabolic process | 10 out of 3118 | 0.76 | 4.85E-05 | NUDT15,GGH,HPRT1,SLC19A1,TPMT,IMPDH2,IMPDH1,XDH,ITPA,GMPS |
| GO:0034641 | cellular nitrogen compound metabolic process | 10 out of 3282 | 0.73 | 7.68E-05 | NUDT15,GGH,HPRT1,SLC19A1,TPMT,IMPDH2,IMPDH1,XDH,ITPA,GMPS |
| GO:0006139 | nucleobase-containing compound metabolic process | 8 out of 2659 | 0.73 | 0.0035 | NUDT15,HPRT1,TPMT,IMPDH2,IMPDH1,XDH,ITPA,GMPS |
| GO:1901564 | organonitrogen compound metabolic process | 9 out of 5244 | 0.48 | 0.0438 | NUDT15,GGH,HPRT1,SLC19A1,IMPDH2,IMPDH1,XDH,ITPA,GMPS |

**Table S8:** Oncogenic nature of mutations predicted using Dr Cancer.

| **Variant ID** | **SNP** | **Method** | | | | | | | | |
| --- | --- | --- | --- | --- | --- | --- | --- | --- | --- | --- |
|  |  | **SEQPROF*** | | | **SVM-GOS**** | | | **SEQPRFGO***** | | |
|  |  | **Probability** | **Reliability Index** | **Prediction** | **Probability** | **Reliability Index** | **Prediction** | **Probability** | **Reliability Index** | **Prediction** |
| rs72552741 | W33G | 0.754 | 5 | Disease | 0.145 | 7 | Neutral | 0.351 | 3 | Neutral |
| rs753277177 | W78R | 0.752 | 5 | Disease | 0.145 | 7 | Neutral | 0.357 | 3 | Neutral |
| rs1784191846 | V89E | 0.777 | 6 | Disease | 0.145 | 7 | Neutral | 0.397 | 2 | Neutral |
| rs1447033392 | W150G | 0.507 | 0 | Disease | 0.145 | 7 | Neutral | 0.27 | 5 | Neutral |
| rs1386533390 | L182P | 0.847 | 7 | Disease | 0.145 | 7 | Neutral | 0.478 | 0 | Neutral |

*SEQPROF : SVM input is the sequence and profile at the mutated position.

**SVM-GOS : SVM input is the GO score for the mutated sequence.

***SEQPRFGO : SVM input is all the input in SEQPROF and GOS.

**Table S9:** Phenotypic consequences of mutations predicted using the FATHMM tool.

| **Variant ID** | **SNP** | **Prediction** | **Score** | **Human Phenotype Ontology Information** |
| --- | --- | --- | --- | --- |
| rs72552741 | W33G | TOLERATED | -0.57 | Phenotypic Abnormality  Abnormality of Head and Neck  Abnormality of The Head  Abnormality of The Face  Abnormality of The Mouth  Abnormality of The Philtrum |
| rs753277177 | W78R | TOLERATED | -1.11 |  |
| rs1784191846 | V89E | TOLERATED | -0.51 |  |
| rs1447033392 | W150G | TOLERATED | -0.73 |  |
| rs1386533390 | L182P | DAMAGING | -1.77 |  |

**Table S10:** Structural effects on TPMT due to mutations, obtained from the HOPE Server.

| Mutation | Amino Acid Alteration | Amino Acid Properties |
| --- | --- | --- |
| W33G | 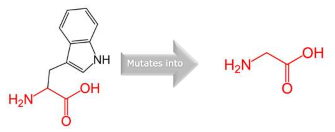 | Glycine is smaller than tryptophan. Hence, the mutation may cause an empty space in the core of the protein.  This mutation introduces a glycine at residue 33 and given that glycine is very flexible, the mutation may disturb the rigidity of the protein at residue 33.  Residue 33 is located within a stretch of residues involved in S-adenosyl-L-methionine binding. Glycine is much more flexible than Tryptophan, hence it can disturb the binding process.  Glycine is less hydrophobic than tryptophan. This may cause a loss of hydrophobic interactions in the core of the protein. |
| W78R | 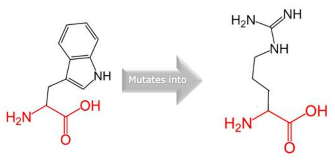 | Arginine is smaller than tryptophan. Hence, the mutation may cause an empty space in the core of the protein.  Tryptophan has a neutral charge, while the mutant residue (Arginine) is positively charged. This charge difference can cause protein folding problems or cause repulsion between the mutant residue and its neighbouring residues.  Arginine is less hydrophobic than tryptophan. This may cause a loss of hydrophobic interactions in the core of the protein. |
| V89E | 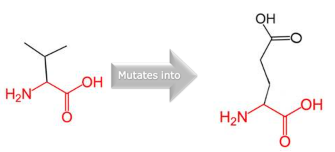 | Since glutamic acid is bigger than valine, it will probably not fit in the core of the protein.  Valine has a neutral charge, while the mutant residue (Glutamic acid) is negatively charged. This charge difference can cause protein folding problems or cause repulsion between the mutant residue and its neighbouring residues.  Residue 89 is located near a stretch of residues involved in S-adenosyl-L-methionine binding. The charge and size differences between valine and glutamic acid may disturb the binding process.  Valine at residue 89 is located in its preferred secondary structure, a β-strand. The mutant residue (Glutamic acid) prefers to be in another secondary structure. Thus, the local conformation will be slightly destabilized.  Glutamic acid is less hydrophobic than valine. This may cause a loss of hydrophobic interactions in the core of the protein. |
| W150G | 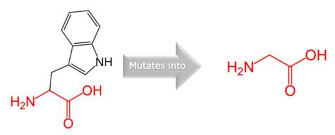 | Glycine is smaller than tryptophan. Hence, the mutation may cause an empty space in the core of the protein.  This mutation introduces a glycine at residue 150 and given that glycine is very flexible, the mutation may disturb the rigidity of the protein at residue 150.  Glycine is less hydrophobic than tryptophan. This may cause a loss of hydrophobic interactions in the core of the protein. |
| L182P | 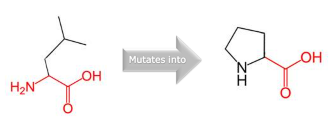 | Proline is smaller than leucine. Hence, the mutation may cause an empty space in the core of the protein. |

**Table S11:** Differences in the binding pockets of the wild-type protein and mutant proteins identified using the CASTp 3.0 server.

| Mutant | Area | |  | Volume | |  | Image of Binding Pocket | |
| --- | --- | --- | --- | --- | --- | --- | --- | --- |
|  | WT | Mutant |  | WT | Mutant |  | WT | Mutant |
| W33G | 562.489 | 664.390 |  | 244.224 | 287.338 |  | 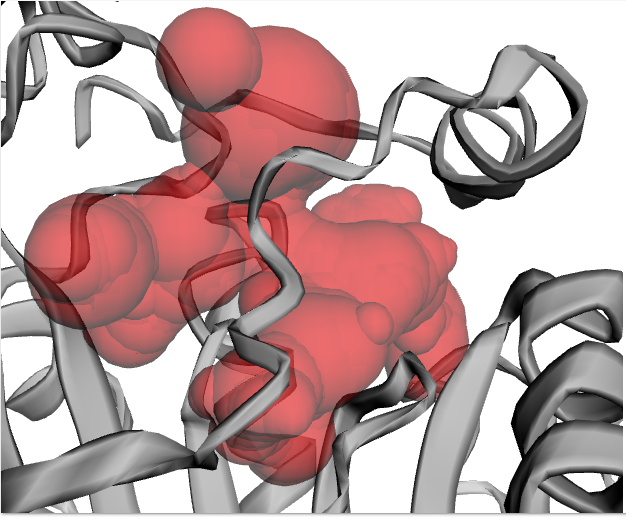 | 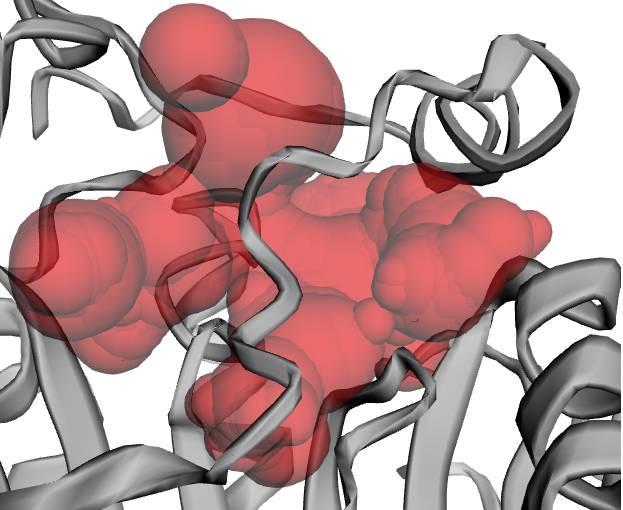 |
| W33G | 1.316 | 0.301 |  | 0.066 | 0.008 |  | 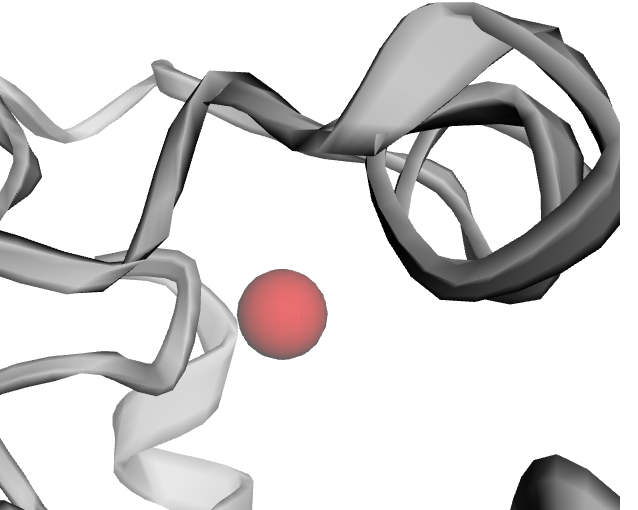 | 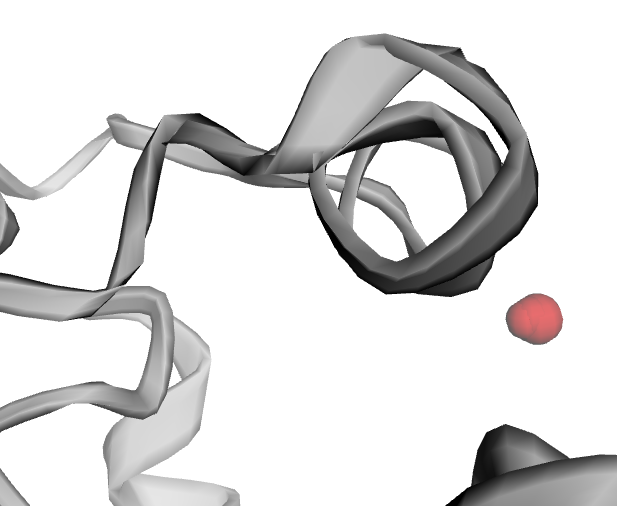 |
| W78R | 7.718 | 6.973 |  | 2.045 | 1.658 |  | 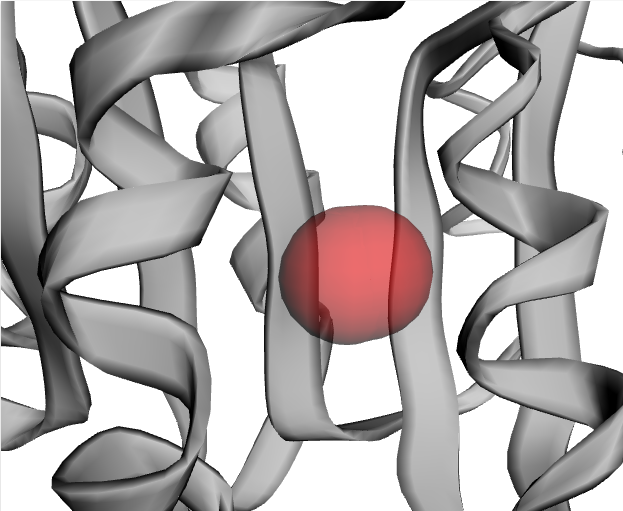 | 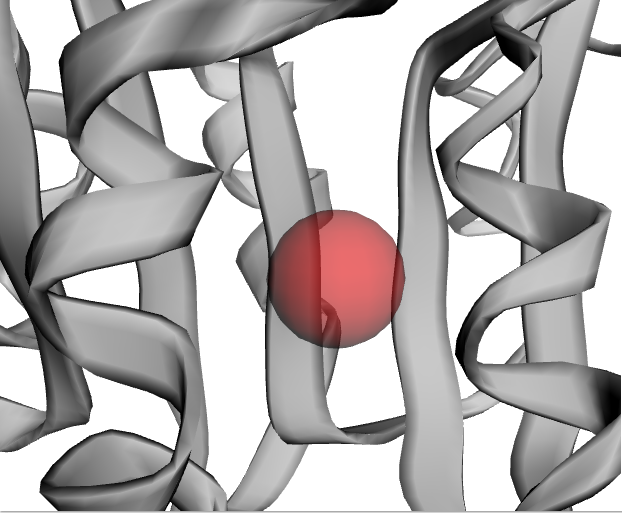 |
| V89E | 562.489 | 562.446 |  | 244.224 | 244.090 |  | 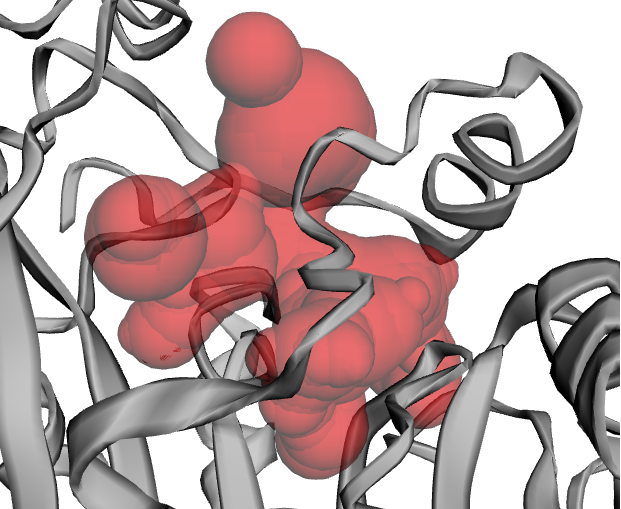 | 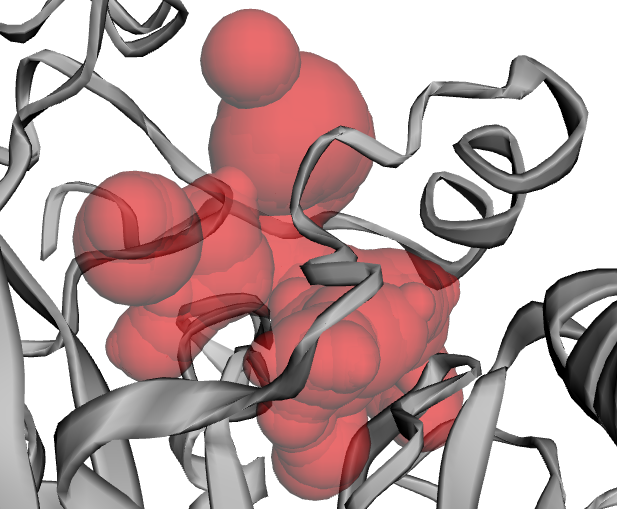 |
| W150G | 562.489 | 662.357 |  | 244.224 | 282.344 |  | 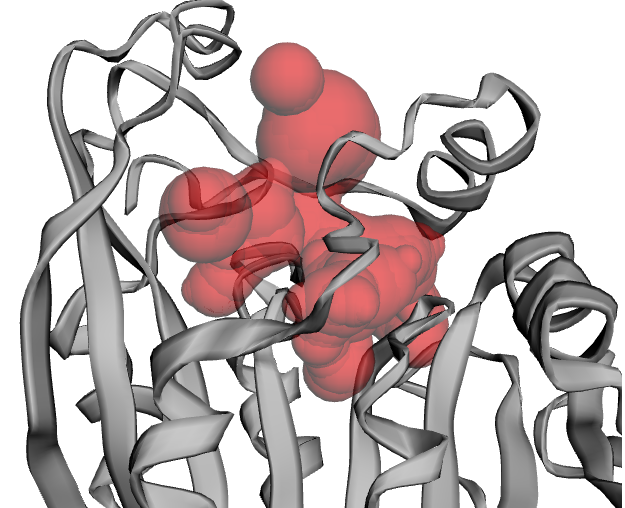 | 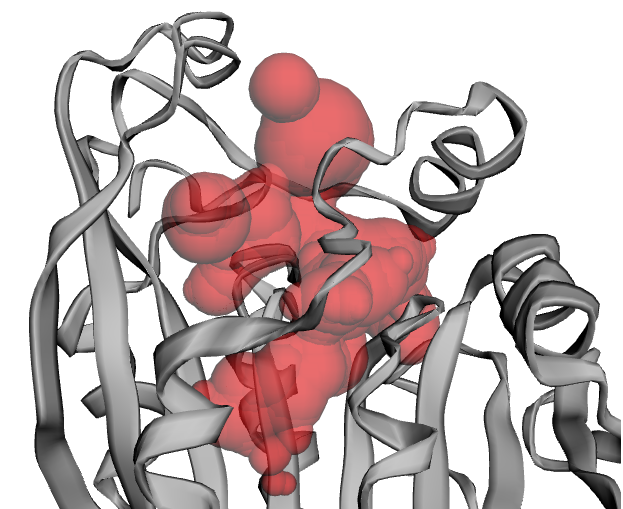 |
| L182P | NA* | 7.200 |  | NA | 0.626 |  | NA | 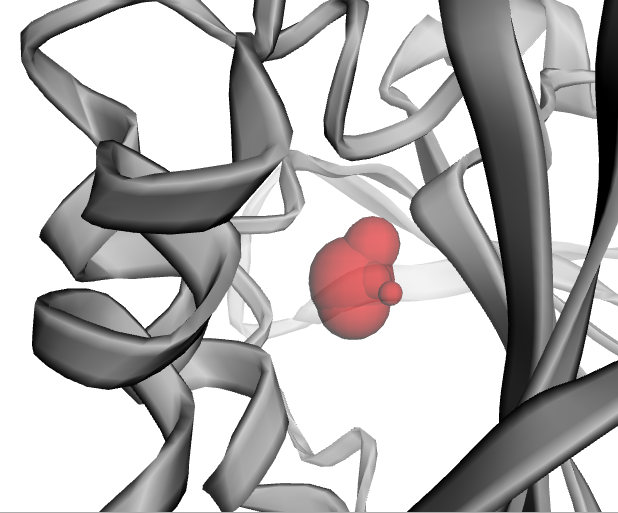 |
| L182P | 0.044 | 3.047 |  | 0.000 | 0.262 |  | 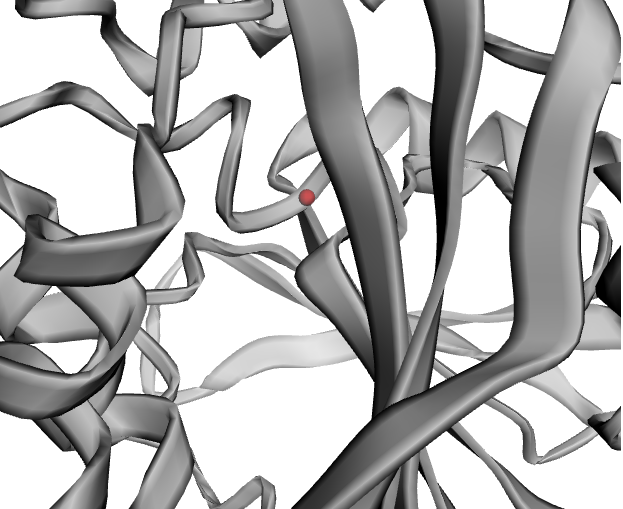 | 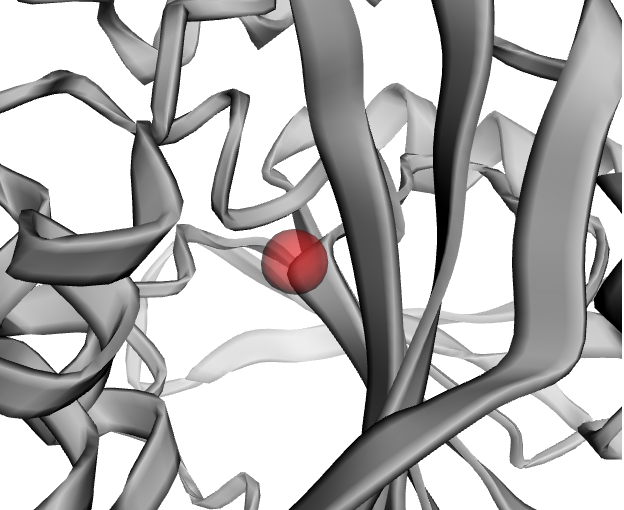 |
| L182P | 0.002 | 0.276 |  | 0.000 | 0.005 |  | 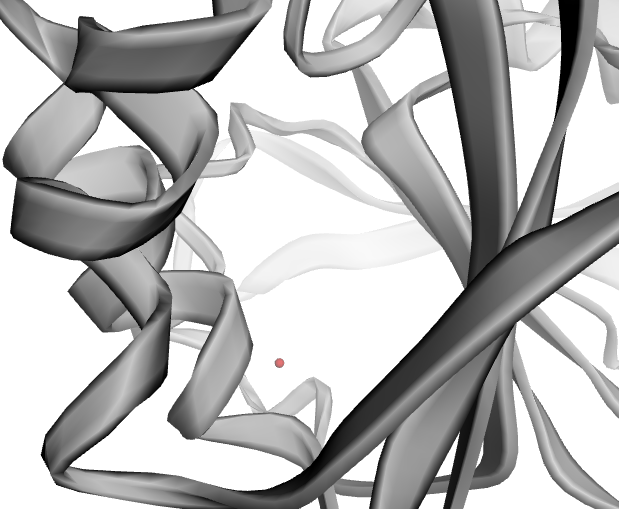 | 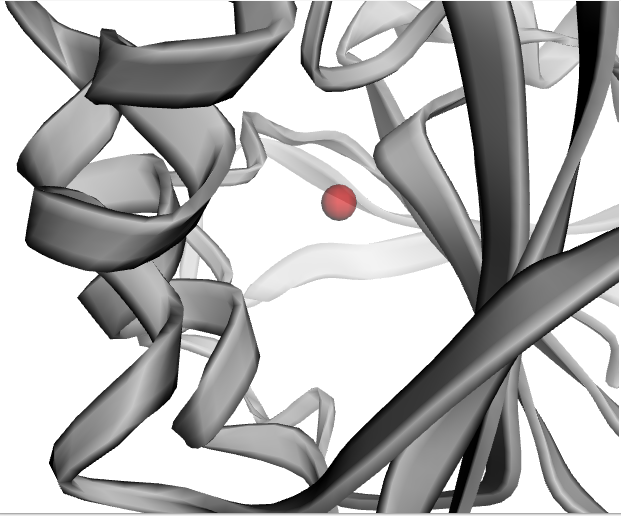 |
| L182P | NA | 0.027 |  | NA | 0.000 |  | NA | 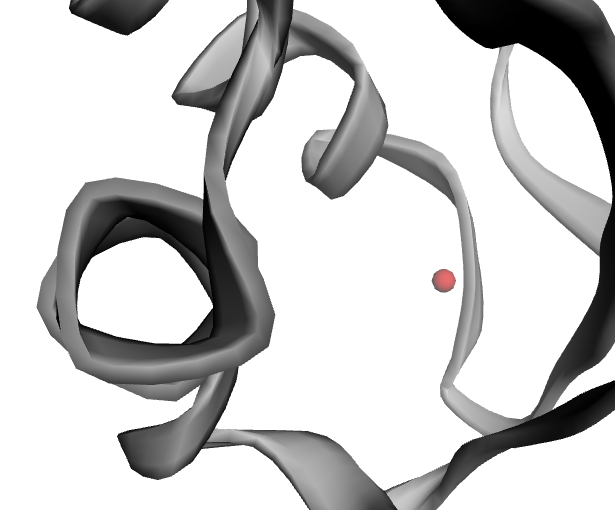 |
| L182P | NA | 0.065 |  | NA | 0.001 |  | NA | 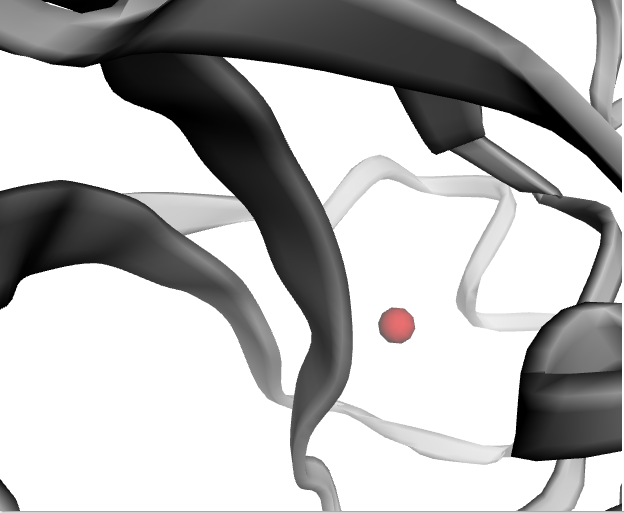 |

*NA=Not applicable as the binding pocket did not form.
