# Supplementary material for: A bioinformatics approach to the identification of novel deleterious mutations of human TPMT through validated screening and molecular dynamics
Source: Sci Rep. 2022 Nov 7;12:18872. doi: 10.1038/s41598-022-23488-z (PMC9640560; doi:10.1038/s41598-022-23488-z)

**ANIMATIONS**

**Animation 1 :** Visualization of the conservation of all residues in chain A of TPMT, obtained from the ConSurf server and visualized through POLYVIEW 3D (The residues corresponding to the 5 screened mutations are highlighted in green).

**
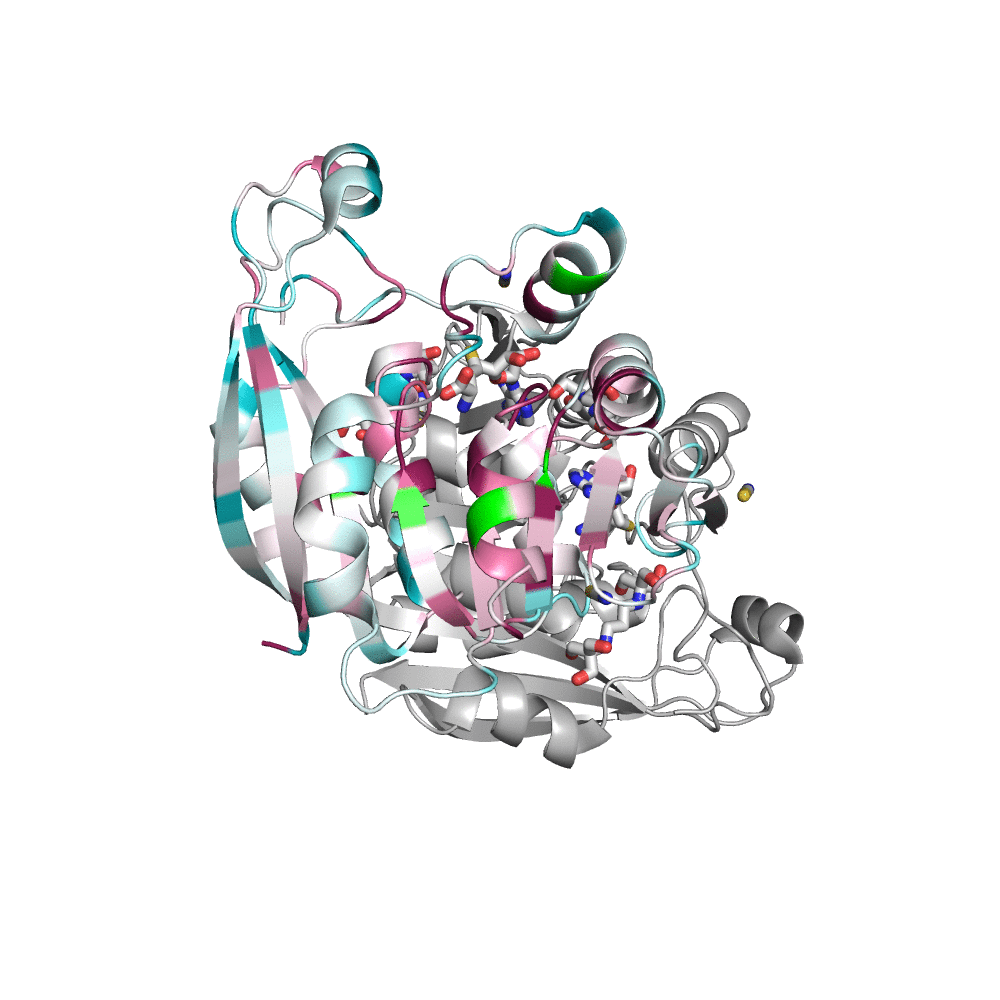
**

**
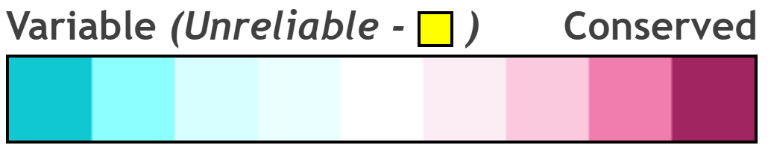
**

**Animation 2 :** Visualization of the 5 screened SNPs, obtained from HOPE Server.

a.) W33G

**
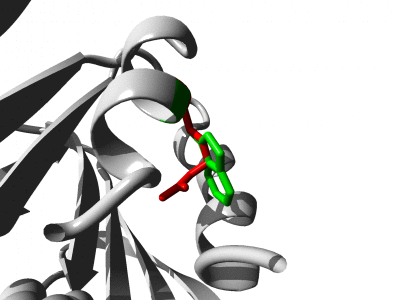
**
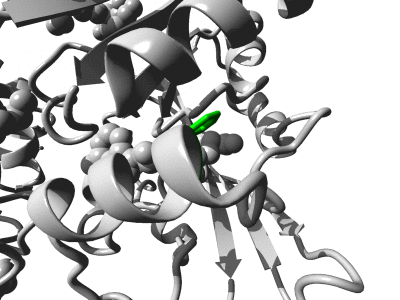


b.) W78R


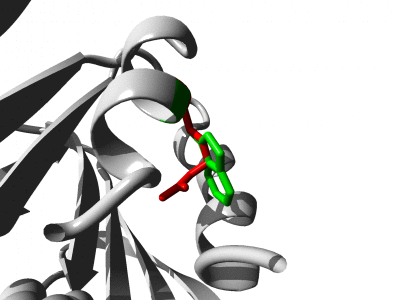

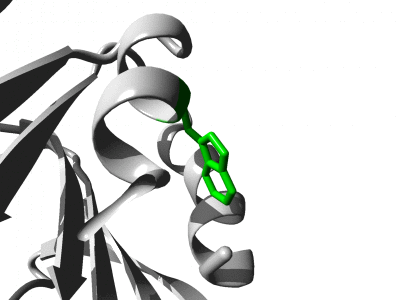


c.) V89E


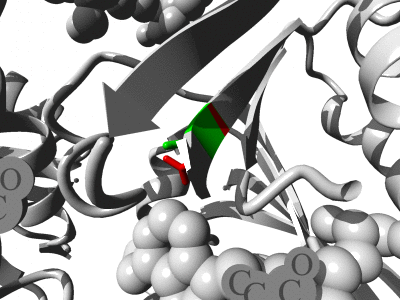

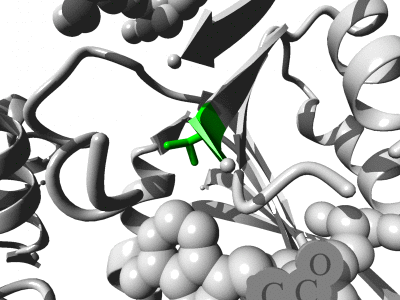


d.) W150G


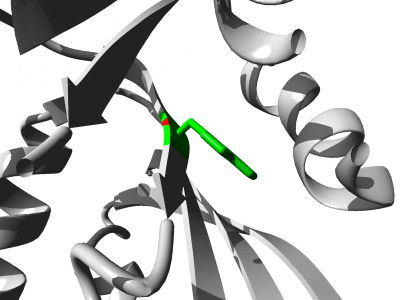

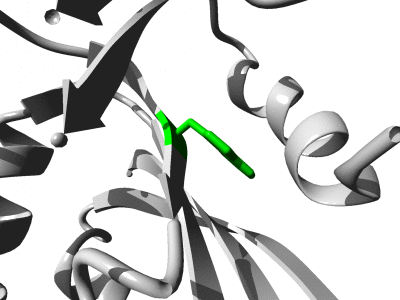


e.) L182P


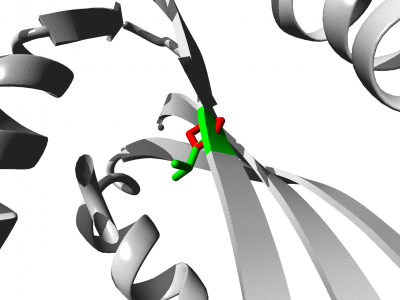

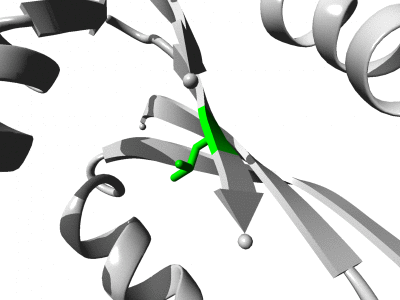

Supplement: Supplementary file 3 — Supplementary Information 3. [file 41598_2022_23488_MOESM3_ESM.docx]
